# Supplementary material for: Counter Regulation of Spic by NF-κB and STAT Signaling Controls Inflammation and Iron Metabolism in Macrophages
Source: Cell Rep. Author manuscript; Available in PMC 2022 Mar 24. (PMC8944937; doi:10.1016/j.celrep.2020.107825)
Supplement: 1 [file NIHMS1608486-supplement-1.pdf]

Cell Reports, Volume *31*

Supplemental Information

Counter Regulation of *Spic* by NF-κB and STAT Signaling Controls Inflammation and Iron Metabolism in Macrophages

Zahidul Alam, Samir Devalaraja, Minghong Li, Tsun Ki Jerrick To, Ian W. Folkert, Erick Mitchell-Velasquez, Mai T. Dang, Patricia Young, Christopher J. Wilbur, Michael A. Silverman, Xinyuan Li, Youhai H. Chen, Paul T. Hernandez, Aritra Bhattacharyya, Mallar Bhattacharya, Matthew H. Levine, and Malay Haldar

Figure S1

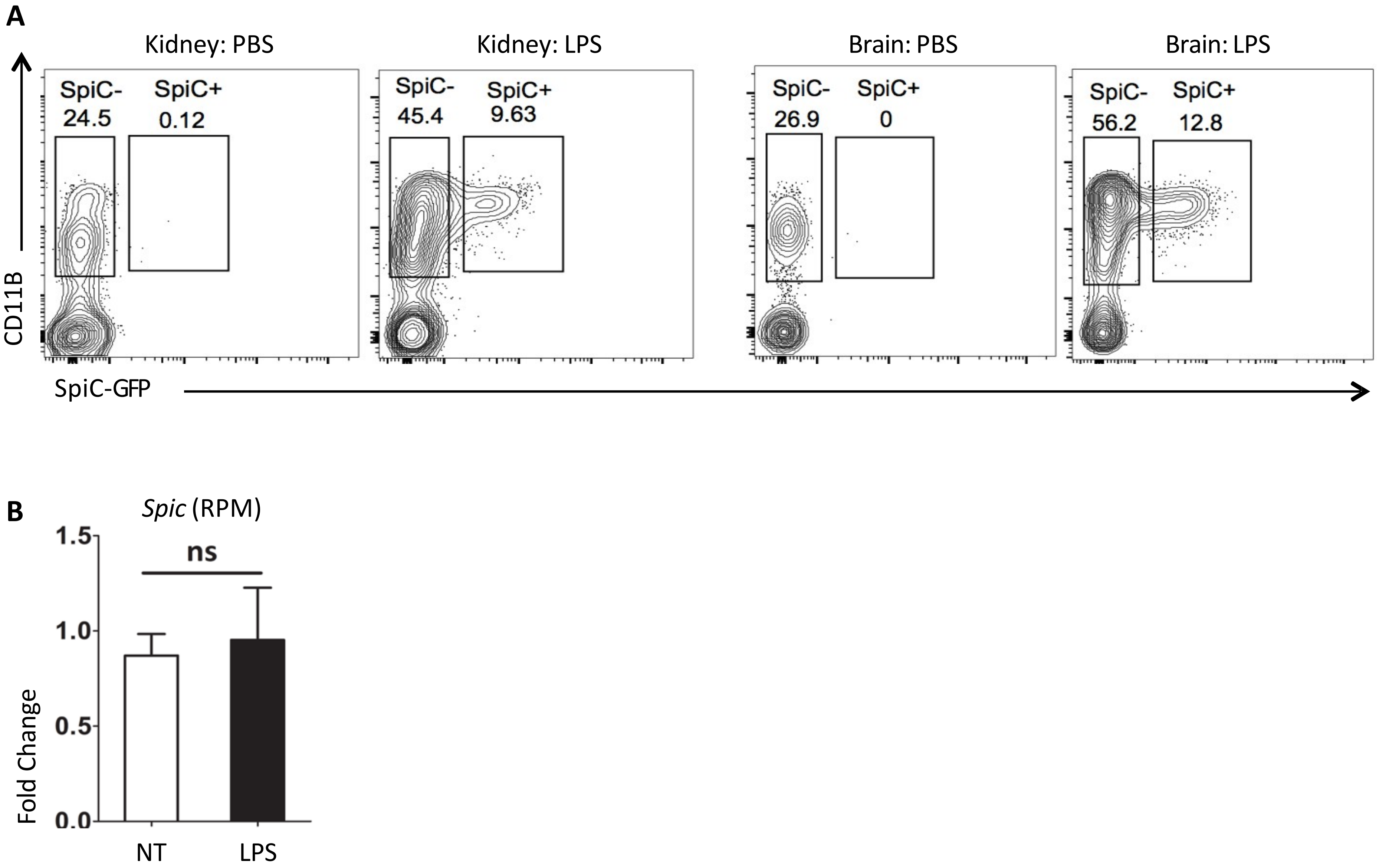
A
Kidney: PBS
Kidney: LPS
Brain: PBS
Brain: LPS
SpiC-
24.5
SpiC+
0.12
SpiC-
45.4
SpiC+
9.63
SpiC-
26.9
SpiC+
0
SpiC-
56.2
SpiC+
12.8
CD11B
SpiC-GFP
B
Spic (RPM)
ns
Fold Change
1.5
1.0
0.5
0.0
NT
LPS

Figure S1. **Related to Figure 1 and 2**

(A) *Spic*$^{GFP/GFP}$ mice treated (i.p) with LPS (75 µg in 200 µl PBS) or control (200 µl PBS) and indicated organs harvested 48 hr. after treatment. Shown are the FCS plots with indicated markers on cells pre-gated for CD45+ singlets. **(B)** *Spic*$^{GFP/GFP}$ mice were treated (i.p) with LPS (7.5 µg/gm body weight). 16 hr. after the treatment, RPMs were isolated purified from spleen *via* FACS, RNA extracted, and the expression (normalized to *Hprt*) of *Spic* measured by qRT-PCR. A trend towards higher *Spic* is observed (P≥0.05). Statistics: unpaired *t*-test. ns: not significant.

FCS: numbers represent percentage of cells within indicated gate. A-D represents ≥3 experiments with ≥3 mice per group. **qRT-PCR**: data representative of ≥3 independent experiments and graphs show single experiment with n≥2 per group. Results expressed as mean ± SEM. NS; not significant.

Figure S2

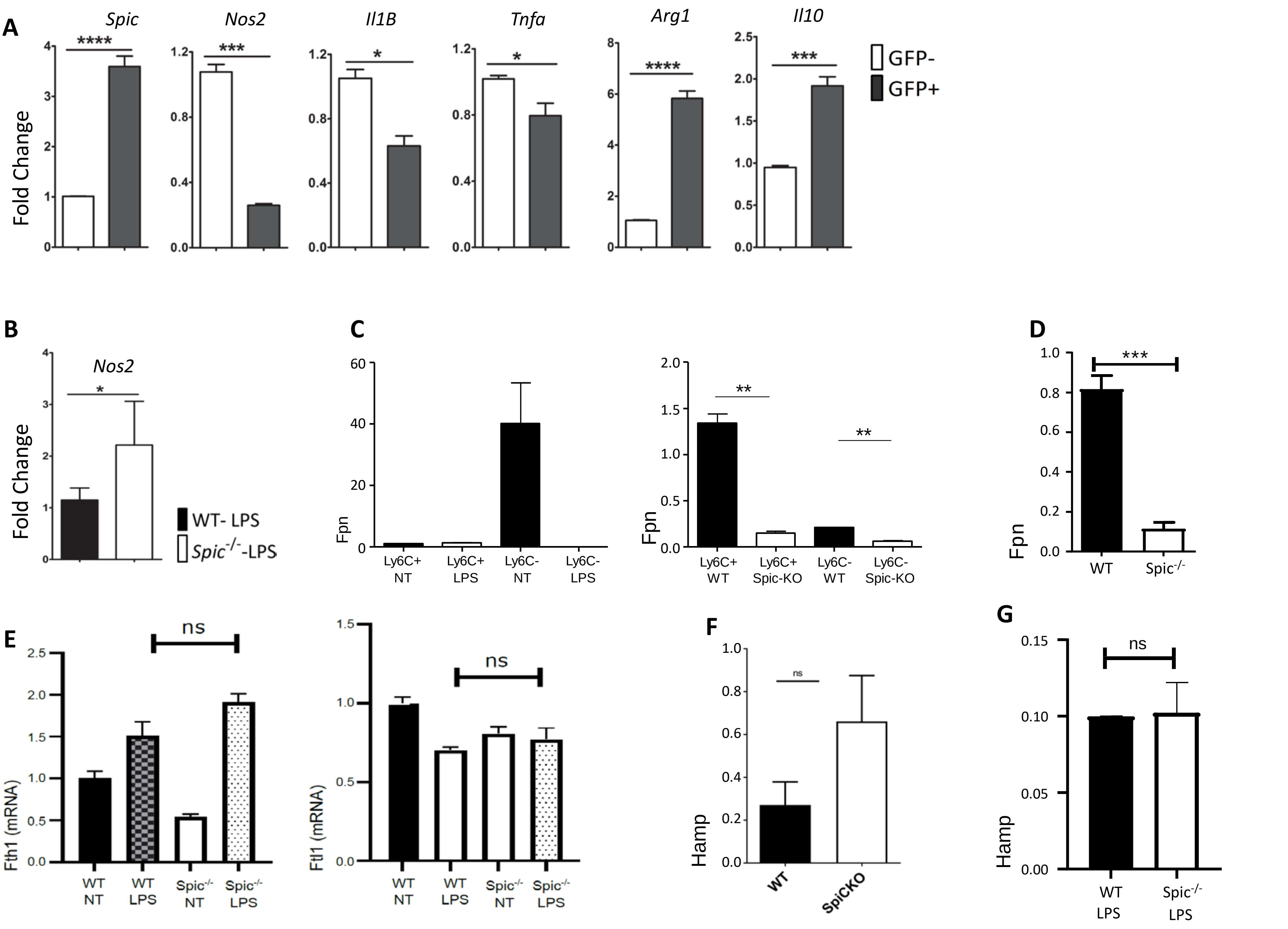

A
Spic
Nos2
Il1B
Tnfa
Arg1
Il10
Fold Change
****
***
*
*
****
***
GFP-
GFP+
B
Nos2
*
Fold Change
WT- LPS
Spic-/--LPS
C
Fpn
Ly6C+ NT
Ly6C+ LPS
Ly6C- NT
Ly6C- LPS
**
**
Ly6C+ WT
Ly6C+ Spic-KO
Ly6C- WT
Ly6C- Spic-KO
D
***
Fpn
WT
Spic-/-
E
ns
Fth1 (mRNA)
WT NT
WT LPS
Spic-/- NT
Spic-/- LPS
ns
Ftl1 (mRNA)
F
ns
Hamp
WT
SpiCKO
G
ns
Hamp
WT LPS
Spic-/- LPS

Figure S2. **Related to Figure 3 and 4**

(A) *Spic*$^{GFP/GFP}$ BMDMs were treated with LPS (1 µg/ml). 48 hr. later, F4/80^{hi}GFP+ and F4/80^{hi}GFP- cells were purified by FACS, re-plated in media and re-exposed to LPS (1 µg/ml). 2 hr. after LPS re-exposure, RNA was extracted and the expression (normalized to *Hprt)* of indicated genes (Y-axis, relative to GFP- cells) measured by qRT-PCR. **(B)** WT or *Spic*$^{-/-}$ mice were treated (i.p) with LPS (150µg per mouse) twice 48 hr. apart. Lungs were harvested 48 hr. after final LPS treatment and the expression of *Nos2* (normalized to *18S rRNA*) measured (relative to expression in non-treated WT lungs) by qRT-PCR. **(C)** Ly6C^{+}TremL4^{-} classical and Ly6C^{-}TremL4^{+} patrolling monocytes were purified (via FACS) from the peripheral blood of three WT and three *Spic*$^{-/-}$ mice at steady state as well as 72 hr. after treatment (i.p) with LPS (150 µg/mouse). Specified monocyte subsets from each genotype were pooled. Shown is the expression (fold change relative to untreated Ly6C^{+} monocytes) of *Fpn* (relative to *Hprt*). The Left panel shows *Fpn* expression with and without LPS treatment in WT mice; showing that LPS-induced reduction in *Fpn* is largely restricted to Ly6C^{-} monocytes. The right panel compares *Fpn* expression upon LPS treatment in WT and *Spic*$^{-/-}$ monocytes, showing that loss of *Spic* reduces *Fpn* in both monocyte subsets. **(D)** Mice of indicated genotype were treated (i.p) with LPS (100 µg/mouse). Peritoneal fluid (all cells, unfractionated) was collected 24 hr later, RNA extracted, and the expression (normalized to *18S* rRNA) of *Fpn* (Y-axis) measured by qRT-PCR. A combination of three independent experiments is shown (3 mice per group). **(E)** WT and *Spic*$^{-/-}$ Mo-MACs were treated with LPS (100 ng/mL). 24 hr. later, RNA was extracted and the expression (normalized to *Hprt*) of Ferritin heavy chain (*Fth1*) and Ferritin light chain (*Ftl1*) measured by qRT-PCR. **(F)** *Spic*$^{-/-}$ (n=3) and WT (n=5) mice were treated with five doses of intraperitoneal LPS (150µg/mouse per dose) 48 hrs. apart. Liver was harvested 24 hr. after the final LPS treatment, RNA extracted and the expression (relative to *Hprt*) of hepcidin (*Hamp*) measured by qRT-PCR. **(G)** WT and *Spic*$^{-/-}$ Mo-MACs were treated with LPS (100 ng/mL). 24 hr. later, RNA was extracted and the expression (normalized to *Hprt*) of *Hamp* measured by qRT-PCR.

qRT-PCR: data representative of ≥3 independent experiments and graphs show single experiment with n≥2 per group. Results expressed as mean ± SEM. $P \leq 0.05$ (*), $P \leq 0.01$ (**), $P \leq 0.001$ (***), and $P \leq 0.0001$ (****), ns; not significant.

Figure S3

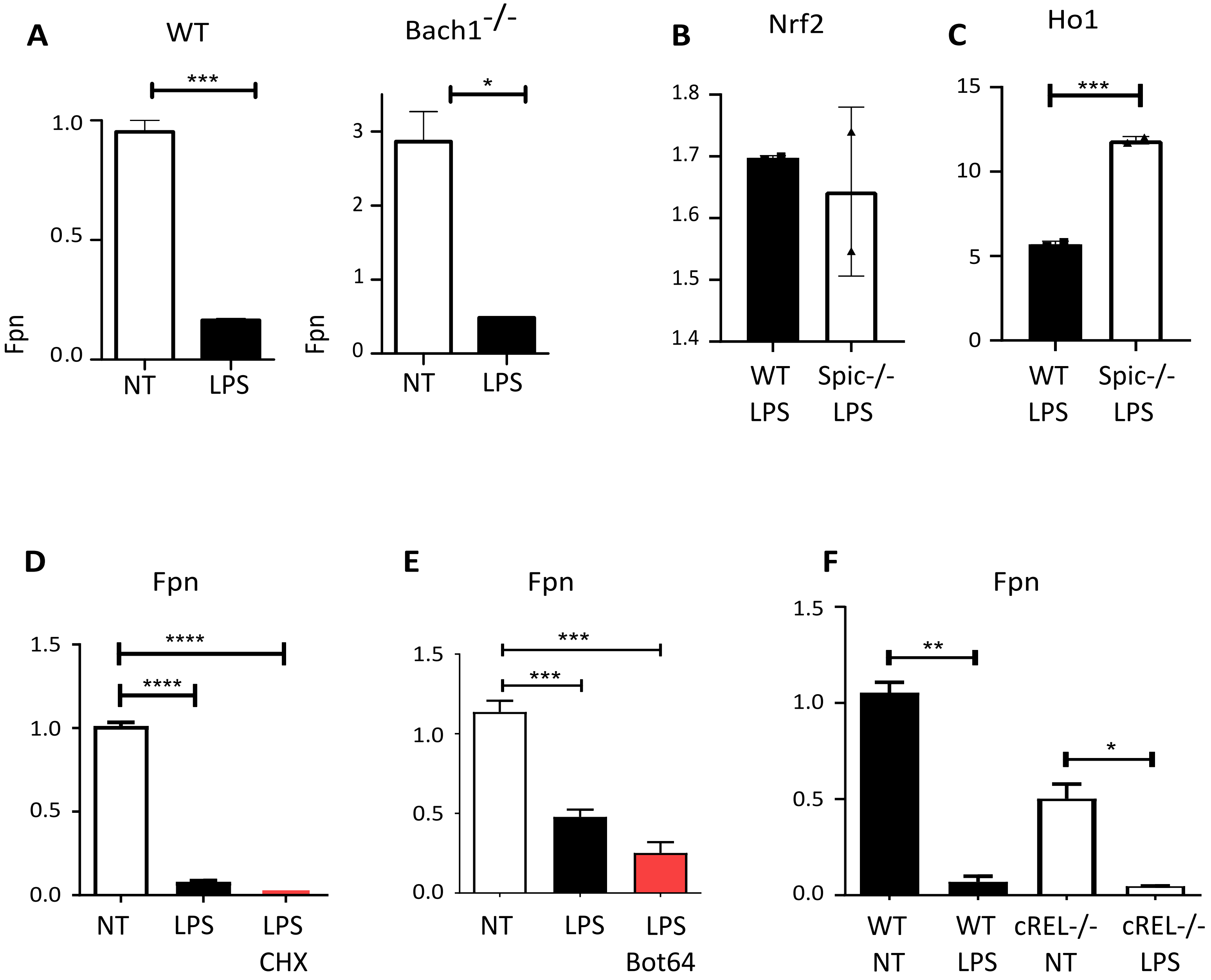
A
WT
Bach1-/-
Fpn
***
*
NT
LPS
B
Nrf2
C
Ho1
WT LPS
Spic-/- LPS
D
E
F
Fpn
****
***
**
LPS CHX
LPS Bot64
WT NT
WT LPS
cREL-/- NT
cREL-/- LPS

Figure S3. **Related to Figure 4**

(A) Mo-MACs of indicated genotypes were treated with LPS (100 ng/mL). 24 hr. later, RNA was extracted and the expression (normalized to *18S*) of *Fpn* measured by qRT-PCR. **(B and C)** Mo-MACs of indicated genotypes were treated with LPS (100 ng/mL). 24 hr. later, RNA was extracted and the expression (normalized to *18S*) of *Fpn* (**B**) and *Ho1* (**C**) measured by qRT-PCR. **(D)** Mo-MACs from WT mice were treated with LPS (1 µg/mL) with or without cycloheximide (10 µg/ml, added one hour before LPS). 8 hr. later, RNA was extracted and the expression (normalized to *Hprt*) of *Fpn* measured by qRT-PCR. **(E)** WT mice were treated (i.p) with LPS (single dose, 100 µg/mouse) with or without Bot64. Bot64 treatment (60 mg/Kg/day, three doses) was started 24 hr. before LPS treatment. 24 hr. after the final Bot64 treatment, mice were euthanized and lungs harvested. Shown is the expression (normalized to *Hprt*) of *Fpn* measured by qRT-PCR. **(F)** Mo-MACs from WT or cREL-knockout mice were treated with LPS (1µg/mL). 16 hr. later, RNA was extracted and the expression of *Fpn* (normalized to *Hprt*) measured by qRT-PCR.

qRT-PCR: data representative of ≥3 independent experiments and graphs show single experiment with n≥2 per group. Results expressed as mean ± SEM. $P \leq 0.05$ (*), $P \leq 0.01$ (**), $P \leq 0.001$ (***), and $P \leq 0.0001$ (****).

Figure S4

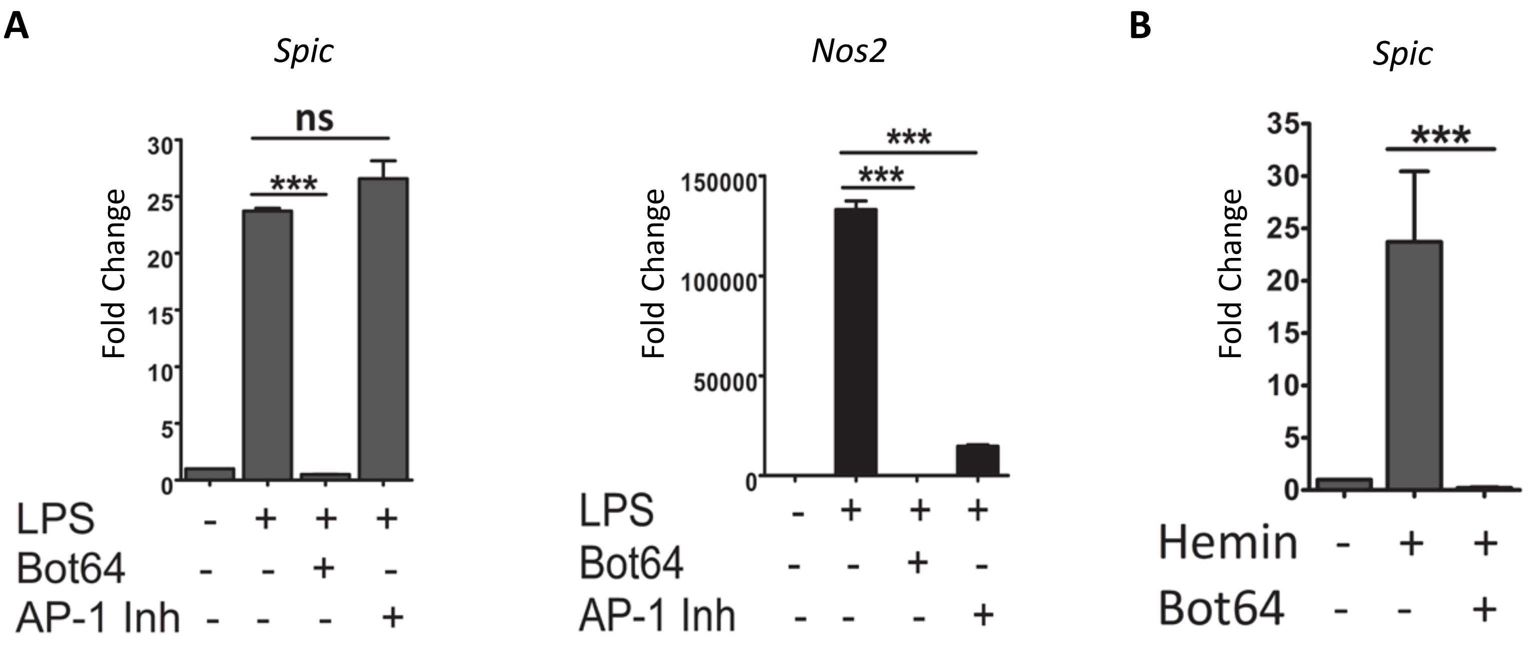

A
Spic
ns
***
Fold Change
30
25
20
15
10
5
0
LPS - + + +
Bot64 - - + -
AP-1 Inh - - - +
Nos2
***
***
Fold Change
150000
100000
50000
0
LPS - + + +
Bot64 - - + -
AP-1 Inh - - - +
B
Spic
***
Fold Change
35
30
25
20
15
10
5
0
Hemin - + +
Bot64 - - +

Figure S4. **Related to Figure 5**

(A) Mo-MACs were treated with LPS (1 µg/ml), IKK-2 inhibitor Bot64 (10 µM), or AP-1 inhibitor T5224 (10 µM). RNA was extracted 20 hr. later and the expression (relative to *Hprt*) of indicated genes (Y-axis, relative to no treatment group) measured by qRT-PCR. AP1 inhibitor blocked *Nos2* induction, but not *Spic*. **(B)** Mo-MACs from WT mice were treated with 80 µM hemin with or without IKK-2 inhibitor (Bot64, 10 µM). RNA was harvested 14 hr. later and the expression (normalized to *Hprt*) of *Spic* measured (Y-axis, relative to no treatment) by qRT-PCR. All qRT-PCR data shown is representative of ≥3 independent experiments. Plots show single experiment with n≥2 per group. To calculate the statistical significance for two individual groups, unpaired *t*-test were performed. To compare the mean of three or more groups, one-way ANOVA with Tukey's multiple comparison tests were used.

qRT-PCR: data representative of ≥3 independent experiments and graphs show single experiment with n≥2 per group. Results expressed as mean ± SEM. $P \le 0.05$ (*), $P \le 0.01$ (**), $P \le 0.001$ (***), and $P \le 0.0001$ (****).

Figure S5

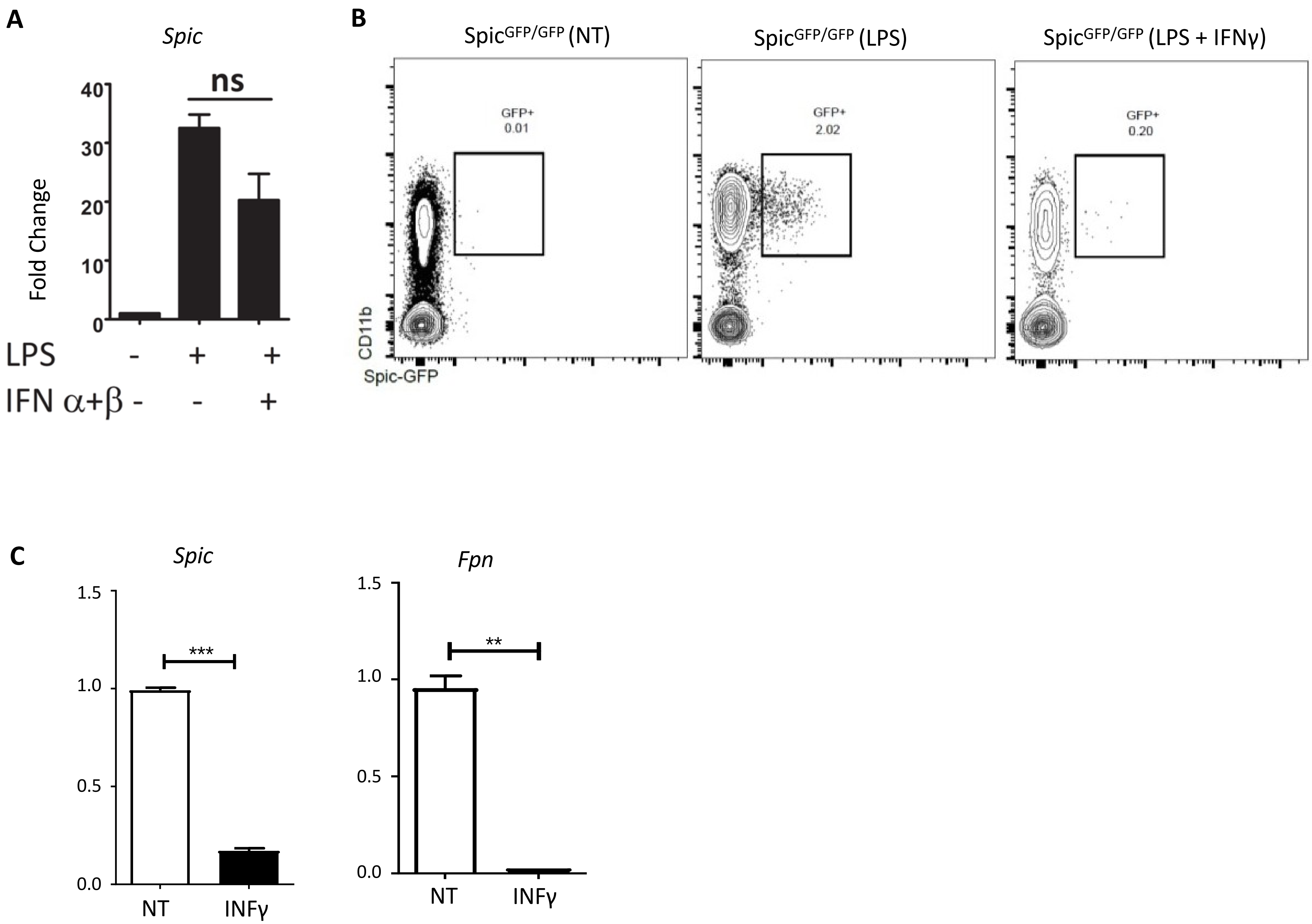
A
Spic
ns
40
30
20
10
0
Fold Change
LPS - + +
IFN α+β - - +
B
SpicGFP/GFP (NT)
SpicGFP/GFP (LPS)
SpicGFP/GFP (LPS + IFNγ)
GFP+
0.01
GFP+
2.02
GFP+
0.20
CD11b
Spic-GFP
C
Spic
1.5
1.0
0.5
0.0
***
NT
INFγ
Fpn
1.5
1.0
0.5
0.0
**
NT
INFγ

Figure S5. **Related to Figure 6**

(A) BMDMs from WT mice were treated with LPS (1 µg/ml) with or without recombinant murine interferon-alpha (IFNα, 50 ng/ml) or interferon-beta (IFNβ, 50 ng/ml). RNA was extracted 18 hr. later and *Spic* expression (normalized to *Hprt*) were measured (Y-axis, fold change compared to no-treatment group) by qRT-PCR. (**B**) $Spic^{GFP/GFP}$ mice were treated with LPS (100µg/mouse) once with or without recombinant interferon-gamma (IFNγ, 20µg/mouse per injection for a total of five injections twice daily). Intraperitoneal IFNγ treatment was started a day before LPS injection. Peripheral blood was taken about 24 hr of LPS treatment. Shown are the flow cytometry plots with indicated markers on cells pre-gated for CD45+Ly6G- singlets. Numbers represent percentage of cells within indicated gate. Data represents 2 or more experiments with 3 or more mice per group. **(C)** BMDMs were treated with LPS (1µg/mL) with or without IFNγ, RNA extracted 20 hr. later, and the expression (normalized to *Hprt*) of *Spic* and *Fpn* measured (Y-axis) by qRT-PCR.

FCS: numbers represent percentage of cells within indicated gate. B represents 2 experiments with ≥3 mice per group. **qRT-PCR**: data representative of ≥3 independent experiments and graphs show single experiment with n≥2 per group. Results expressed as mean ± SEM. $P \leq 0.05$ (*), $P \leq 0.01$ (**), $P \leq 0.001$ (***), and $P \leq 0.0001$ (****).

Figure S6

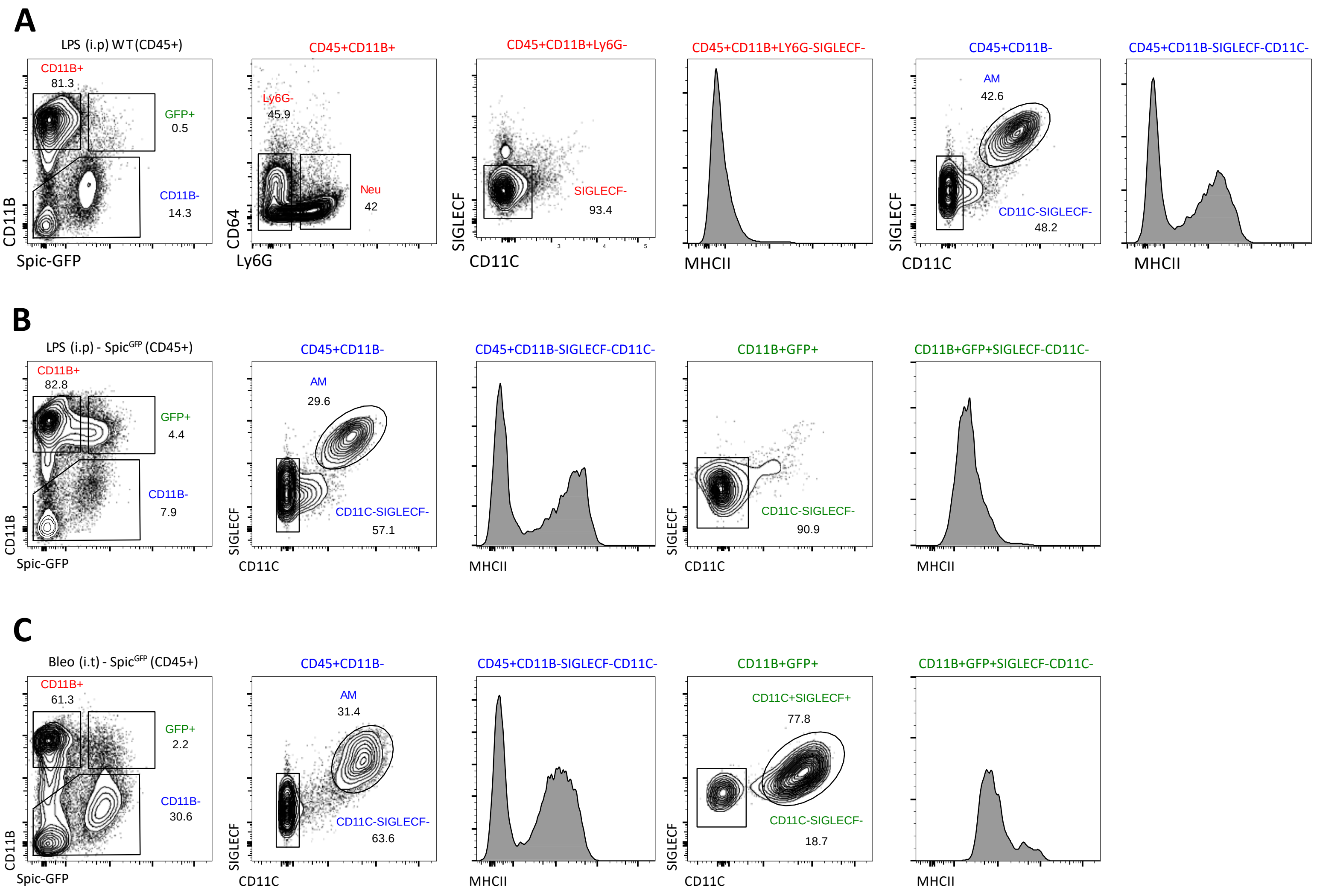
A
LPS (i.p) WT(CD45+)
CD11B+
81.3
GFP+
0.5
CD11B-
14.3
CD11B
Spic-GFP
CD45+CD11B+
Ly6G-
45.9
Neu
42
CD64
Ly6G
CD45+CD11B+Ly6G-
SIGLECF-
93.4
SIGLECF
CD11C
CD45+CD11B+LY6G-SIGLECF-
MHCII
CD45+CD11B-
AM
42.6
CD11C-SIGLECF-
48.2
SIGLECF
CD11C
CD45+CD11B-SIGLECF-CD11C-
MHCII
B
LPS (i.p) - Spic^GFP (CD45+)
CD11B+
82.8
GFP+
4.4
CD11B-
7.9
CD11B
Spic-GFP
CD45+CD11B-
AM
29.6
CD11C-SIGLECF-
57.1
SIGLECF
CD11C
CD45+CD11B-SIGLECF-CD11C-
MHCII
CD11B+GFP+
CD11C-SIGLECF-
90.9
SIGLECF
CD11C
CD11B+GFP+SIGLECF-CD11C-
MHCII
C
Bleo (i.t) - Spic^GFP (CD45+)
CD11B+
61.3
GFP+
2.2
CD11B-
30.6
CD11B
Spic-GFP
CD45+CD11B-
AM
31.4
CD11C-SIGLECF-
63.6
SIGLECF
CD11C
CD45+CD11B-SIGLECF-CD11C-
MHCII
CD11B+GFP+
CD11C+SIGLECF+
77.8
CD11C-SIGLECF-
18.7
SIGLECF
CD11C
CD11B+GFP+SIGLECF-CD11C-
MHCII

Figure S6. **Related to Figure 7 and 2**

(A) WT mice were treated (i.p) with LPS (150 µg/mouse), euthanized 48 hr. later, and their lungs analyzed by FCS using indicated markers. Headers denote pre-gating. The initial gates (first panel) are color-coded. Alveolar macrophages (AM, fifth panel) and the two subsets (MHC-II+ and MHC II-) of interstitial macrophages (IM, fifth and sixth panel) are clearly distinguishable. **(B)** *Spic*$^{GFP/GFP}$ reporter mice were treated (i.p) with LPS (150 µg/mouse) and euthanized 48 hr. later. Shown are the FCS plots of lungs using the gating strategy described in (A). AMs (second panel) and the two subsets of IMs (second and third panel) are clearly distinguishable. SPIC (GFP+) expressing cells (fourth and fifth panel) resemble MHCII+ IMs (fourth and fifth panel). **(C)** *Spic*$^{GFP/GFP}$ reporter mice were treated with intra-tracheal (i.t) bleomycin (3 U/kg in 50µl sterile PBS) and euthanized four weeks later. Shown are the FCS plots using the gating strategy described in (A). AMs (second panel) and the two subsets of IMs (third panel) are clearly distinguishable. In contrast to LPS treatment, SPIC (GFP+) expressing cells (fourth and fifth panel) appear to contain both AMs and the two subsets of IMs.

FCS: numbers represent percentage of cells within indicated gate. Data representative of ≥ 3 experiments with ≥ 3 mice per group per experiment.

Figure S7

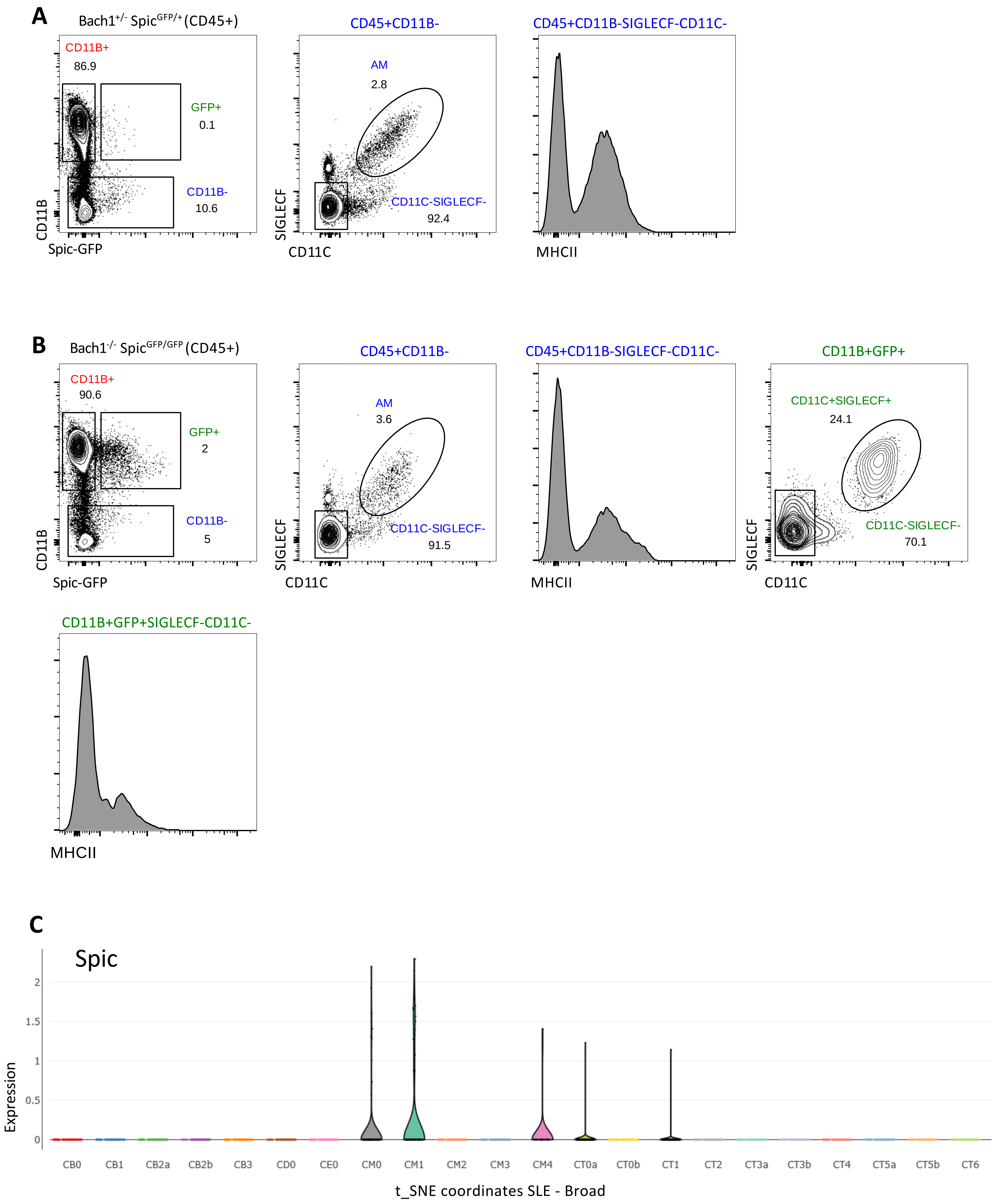
A
Bach1+/- SpicGFP/+ (CD45+)
CD11B+
86.9
GFP+
0.1
CD11B-
10.6
CD11B
Spic-GFP
CD45+CD11B-
AM
2.8
CD11C-SIGLECF-
92.4
SIGLECF
CD11C
CD45+CD11B-SIGLECF-CD11C-
MHCII
B
Bach1-/- SpicGFP/GFP (CD45+)
CD11B+
90.6
GFP+
2
CD11B-
5
CD11B
Spic-GFP
CD45+CD11B-
AM
3.6
CD11C-SIGLECF-
91.5
SIGLECF
CD11C
CD45+CD11B-SIGLECF-CD11C-
MHCII
CD11B+GFP+
CD11C+SIGLECF+
24.1
CD11C-SIGLECF-
70.1
SIGLECF
CD11C
CD11B+GFP+SIGLECF-CD11C-
MHCII
C
Spic
Expression
0
0.5
1
1.5
2
CB0
CB1
CB2a
CB2b
CB3
CD0
CE0
CM0
CM1
CM2
CM3
CM4
CT0a
CT0b
CT1
CT2
CT3a
CT3b
CT4
CT5a
CT5b
CT6
t_SNE coordinates SLE - Broad

Figure S7. Related to Figure 7 and 2

(A) Lungs from *Bach1*$^{+/-}$ *Spic*$^{GFP/+}$ mice were analyzed by FCS using the indicated markers. The gates are color-coded and very few GFP + *Spic*-expressing cells are seen (first panel). AMs (middle panel) and the two subsets of IMs (last panel) are identified.

(B) Lungs from *Bach1*$^{-/-}$ *Spic*$^{GFP/GFP}$ mice were analyzed by FCS using the indicated markers. The gates are color-coded and highlights the presence of GFP+ *Spic*-expressing cells in the absence of *Bach1* (first panel). AMs (second panel) and the two subsets of IMs (third panel) are identified. SPIC (GFP+) expressing cells (fourth and fifth panel) appear to contain both AMs and the two subsets of IMs.

FCS: numbers represent percentage of cells within indicated gate. Data representative of ≥ 3 experiments with ≥ 3 mice per group per experiment

(C) The expression of Spic was examined in single cell RNA-sequencing dataset from Kidneys of normal humans and patients of lupus nephritis (Arazi et al., 2019). This data was generated using the freely available single cell portal from the broad institute (https://singlecell.broadinstitute.org/single_cell/study/SCP279/amp-phase-1) using their lupus nephritis dataset. Shown is the expression of Spic (Y-axis) in various kidney-infiltrating immune cells (X-axis).

As described in detail by Arazi and colleagues, cluster CM0 to CM4 were identified as myeloid cells. CM0, CM1, and CM4 were identified as kidney-infiltrating monocytes and macrophages as these cells were enriched in nephritic kidneys compared to normal kidneys. Of these, CM1 and CM4 were identified as phagocytic macrophages. CM4 was further characterized as alternatively activated macrophages with high expression of ferroportin. Further analyses by Arazi and colleagues uncovered a differentiation trajectory where blood monocytes (CM0) differentiate into phagocytic monocyte/macrophage (CM1), which further differentiates into an alternatively activated macrophage (CM4) in nephritic kidneys (Arazi et al., 2019). In contrast, CM2 was the dominant myeloid cells in normal kidneys and characterized as tissue-resident macrophages (Arazi et al., 2019). The relatively specific expression of Spic within the inflammation-associated monocyte-derived lineage (CM0, CM1, and CM4), but not in dendritic cells (CM3) or tissue-resident macrophages (CM2), is consistent with our observations in murine inflammation models.

CM0: Patrolling CD16+ macrophages, CM1: Phagocytic CD16+ macrophages, CM2: Tissue-resident macrophages, CM3: cDCs, CM4: M2-like CD16+ macrophages, CT0a: Effector memory CD4+ T cells, CT0b: Central memory CD4+ T cells, CT1: CD56dimCD16+ NK cells, CT2: CTLs, CT3a: Treg cells, CT3b: TFH-like cells, CT4: GZMK+ CD8+ T cells, CT5a: Resident memory CD8+ T cells, CT5b: CD56brightCD16– NK cells, CT6: ISG-high CD4+ T cells, CB0: Activated B cells, CB1: Plasma, cells and plasmablasts, CB2a: Naive B cells, CB2b: pDCs, CB3: ISG-high B cells, CD0: Dividing cells, CE0: Epithelial cells.
