# Supplementary material for: Counter Regulation of Spic by NF-κB and STAT Signaling Controls Inflammation and Iron Metabolism in Macrophages
Source: Cell Rep. Author manuscript; Available in PMC 2022 Mar 24. (PMC8944937; doi:10.1016/j.celrep.2020.107825)
Supplement: 2 [file NIHMS1608486-supplement-2.pdf]

Cell Reports

Article

Counter Regulation of *Spic* by NF-κB and STAT Signaling Controls Inflammation and Iron Metabolism in Macrophages

Graphical Abstract

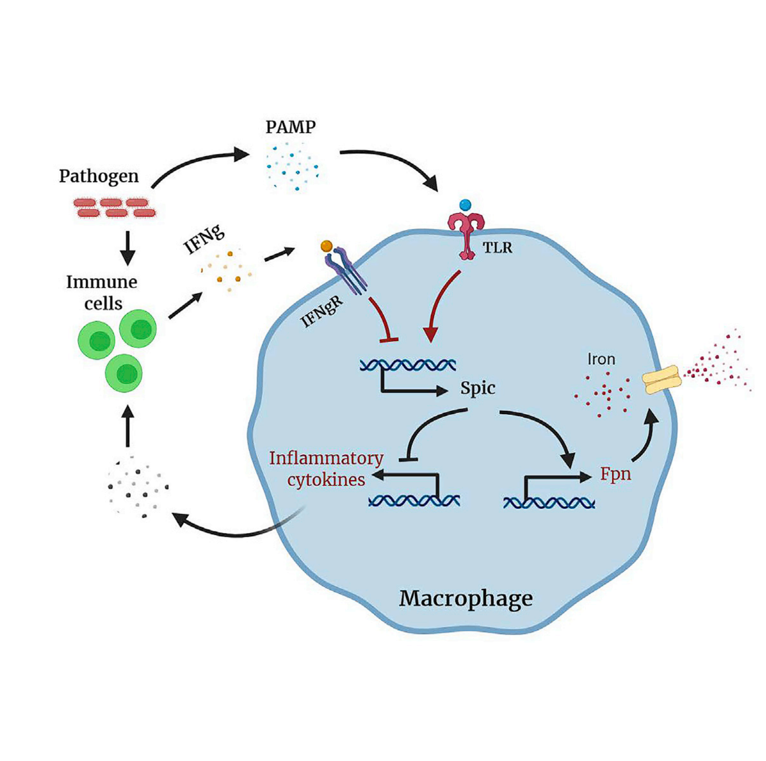

Highlights

- The transcription factor *Spic* restrains inflammatory responses in macrophages
- *Spic* promotes the expression of the iron exporter ferroportin in activated macrophages
- NF-κB activity is required for the expression of *Spic* in activated macrophages
- Interferon-gamma suppresses *Spic* expression in activated macrophages

Authors

Zahidul Alam, Samir Devalaraja, Minghong Li, ..., Mallar Bhattacharya, Matthew H. Levine, Malay Haldar

Correspondence

mhaldar@pennmedicine.upenn.edu

In Brief

Activated macrophages must fine-tune their inflammatory responses to promote host defense while limiting tissue damage. Alam et al. find that the transcription factor *Spic* restrains inflammatory responses and promotes iron efflux from activated macrophages, thereby calibrating macrophage responses during the resolution of inflammation.

Alam et al., 2020, Cell Reports *31*, 107825
June 30, 2020 © 2020 The Author(s).
https://doi.org/10.1016/j.celrep.2020.107825

CellPress

Cell Reports

CellPress
OPEN ACCESS

Article

Counter Regulation of *Spic* by NF-κB and STAT Signaling Controls Inflammation and Iron Metabolism in Macrophages

Zahidul Alam,[1] Samir Devalaraja,[1] Minghong Li,[1] Tsun Ki Jerrick To,[1] Ian W. Folkert,[2] Erick Mitchell-Velasquez,[1] Mai T. Dang,[3] Patricia Young,[1,3] Christopher J. Wilbur,[4,5] Michael A. Silverman,[4,5] Xinyuan Li,[1,5] Youhai H. Chen,[1,5] Paul T. Hernandez,[2] Aritra Bhattacharyya,[6] Mallar Bhattacharya,[6] Matthew H. Levine,[2] and Malay Haldar[1,5,7,8,*]

[1]Department of Pathology and Laboratory Medicine, Perelman School of Medicine, University of Pennsylvania, PA 19104, USA
[2]Department of Surgery, Perelman School of Medicine, University of Pennsylvania, PA 19104, USA
[3]Department of Neurology, The Children's Hospital of Philadelphia, Perelman School of Medicine, University of Pennsylvania, PA 19104, USA
[4]Department of Pediatrics, The Children's Hospital of Philadelphia, Perelman School of Medicine, University of Pennsylvania, PA 19104, USA
[5]Institute for Immunology, Perelman School of Medicine, University of Pennsylvania, PA 19104, USA
[6]Division of Pulmonary, Critical Care, Allergy, and Sleep Medicine, Department of Medicine, University of California, San Francisco, San Francisco, CA, USA
[7]Abramson Family Cancer Research Institute, Perelman School of Medicine, University of Pennsylvania, PA 19104, USA
[8]Lead Contact
*Correspondence: mhaldar@pennmedicine.upenn.edu
https://doi.org/10.1016/j.celrep.2020.107825

SUMMARY

Activated macrophages must carefully calibrate their inflammatory responses to balance efficient pathogen control with inflammation-mediated tissue damage, but the molecular underpinnings of this "balancing act" remain unclear. Using genetically engineered mouse models and primary macrophage cultures, we show that Toll-like receptor (TLR) signaling induces the expression of the transcription factor *Spic* selectively in patrolling monocytes and tissue macrophages by a nuclear factor κB (NF-κB)-dependent mechanism. Functionally, *Spic* downregulates pro-inflammatory cytokines and promotes iron efflux by regulating ferroportin expression in activated macrophages. Notably, interferon-gamma blocks *Spic* expression in a STAT1-dependent manner. High levels of interferon-gamma are indicative of ongoing infection, and in its absence, activated macrophages appear to engage a "default" *Spic*-dependent anti-inflammatory pathway. We also provide evidence for the engagement of this pathway in sterile inflammation. Taken together, our findings uncover a pathway wherein counter-regulation of *Spic* by NF-κB and STATs attune inflammatory responses and iron metabolism in macrophages.

INTRODUCTION

Macrophages are widely distributed with impressive functional diversity (Gordon et al., 2014; Haldar and Murphy, 2014). At steady state, tissue macrophages help maintain local tissue homeostasis (Gordon et al., 2014; Haldar and Murphy, 2014). Injury or infection leads to the recruitment of circulating monocytes that can locally differentiate into macrophages (monocyte-derived macrophages [Mo-MACs]) that produce cytokines and other factors that shape the ensuing immune response. Resolution of inflammation is facilitated by reduced pro-inflammatory and increased anti-inflammatory cytokine production by Mo-MACs (Murray, 2017; Oishi and Manabe, 2018; Wynn and Vannella, 2016). Excessive or prolonged inflammatory responses can impair tissue repair, whereas suboptimal responses lead to poor pathogen control (Murray and Wynn, 2011). Therefore, macrophage inflammatory responses are dynamically regulated, but the molecular underpinnings are unclear.

Macrophages have receptors that detect pathogen-associated or endogenous danger-associated molecular patterns (PAMPs and DAMPs, respectively) (Mukhopadhyay et al., 2009; Zhang and Mosser, 2008). Lipopolysaccharide (LPS), the prototypical PAMP, activates Toll-like receptor 4 (TLR4), which induces large-scale transcriptional changes. LPS-induced genes can be classified as primary and secondary response genes (Medzhitov and Horng, 2009). The induction of primary response genes does not require new protein synthesis and occurs within minutes by activation of pre-existing transcription factors, such as nuclear factor κB (NF-κB) and AP-1. Primary response genes mainly promote inflammation; however, a subset of these genes encode transcription factors that mediate the expression of secondary response genes with more diverse function (Medzhitov and Horng, 2009). Temporally and mechanistically, the regulation of secondary response genes provides a convenient fulcrum for calibrating macrophage inflammatory responses. As an example, the transcription factor C/EBPβ is a secondary response gene that counteracts the pro-inflammatory

This is an open access article under the CC BY license (http://creativecommons.org/licenses/by/4.0/).

actions of NF-κB (Kaneda et al., 2016; Ruffell et al., 2009). Nonetheless, how secondary response genes regulate macrophage inflammatory responses is not fully understood.

PAMP recognition induces cytokine production by macrophages that alert the rest of the immune system to the presence of pathogens. The subsequent influx and activation of other immune cells at the site of inflammation change the cytokine milieu. Sensing this evolving cytokine milieu is one way through which macrophages can assess the status of local inflammation to regulate their own function accordingly (Oishi and Manabe, 2018; Wynn and Vannella, 2016). T cells and natural killer (NK) cells produce high levels of interferon-gamma (IFNγ) at sites of infection, which dissipates upon resolution of infection (Thäle and Kiderlen, 2005). Hence, IFNγ can serve as a "second signal" for PAMP-activated macrophages, corroborating the presence of pathogens. Consistent with this notion, IFNγ augments inflammatory and microbicidal functions of macrophages (Hu and Ivashkiv, 2009). However, how activated macrophages respond to falling IFNγ levels in the resolution phase of pathogen-induced inflammation and how IFNγ affects macrophage function during sterile inflammation remain unclear.

Iron enters macrophages through diverse pathways and is either stored inside the cell or released back into the surrounding environment by the iron exporter ferroportin (Fpn, Slc40a1) (Alam et al., 2017; Soares and Hamza, 2016). Because pathogens require iron to thrive in the host, macrophages sequester it during infection (Ganz and Nemeth, 2015). A key mechanism controlling iron availability is the regulation of macrophage *Fpn* during inflammation (Drakesmith et al., 2015). Toll-like receptor (TLR) activation rapidly downregulates *Fpn* transcription (Guida et al., 2015). Hepcidin, a peptide hormone produced by hepatocytes during inflammation, also causes internalization and degradation of FPN in macrophages (Drakesmith and Prentice, 2012). Systemically, prolonged or excessive iron sequestration can lead to iron deficiency, whereas locally this can impair wound repair (Ganz and Nemeth, 2015; Recalcati et al., 2019). Therefore, macrophages must release trapped iron during the resolution phase of inflammation, but pathways linking the resolution of inflammation to iron efflux in macrophages are unclear.

The transcription factor *Spic* was previously shown to be required for the development of iron-recycling macrophages (Haldar et al., 2014; Kohyama et al., 2009). Here, we show that *Spic* is also induced in PAMP- or DAMP-activated macrophages where it reduces the inflammatory response and promotes iron efflux. In this setting, the mechanism of *Spic* induction is distinct from the previously reported *Bach1* and heme-dependent pathway. Importantly, IFNγ blocks *Spic* expression, providing insight into how *Spic*-dependent functions are differentially engaged in the presence or absence of an infectious threat.

RESULTS

TLR Ligands Selectively Induce *Spic* Expression in Patrolling Monocytes and Tissue Macrophages

Spic regulates the development of iron-recycling macrophages in the spleen and bone marrow (Haldar et al., 2014; Kohyama et al., 2009). Because monocytes and macrophages alter iron metabolism during inflammation (Ganz and Nemeth, 2015), we examined whether inflammation regulates *Spic* by treating *Spic*$^{GFP/GFP}$ reporter mice (Haldar et al., 2014) with intraperitoneal LPS. In the blood, LPS induced *Spic* selectively in monocytes (Figures 1A–1C). Ly6C expression marks two major subsets of murine monocytes (Geissmann et al., 2003). Notably, *Spic* was induced in Ly6C^{lo}TremL4^{hi} (patrolling) but not Ly6C^{hi}TremL4^{lo} (classical) monocytes (Figures 1B and 1C). To test whether blood monocyte subsets inherently differ in their capacity to express *Spic*, we analyzed *Spic* expression in *Bach1*-deficient *Spic*$^{GFP/GFP}$ reporter (*Bach1*$^{-/-}$: *Spic*$^{GFP/GFP}$) mice. We previously showed that the transcription factor *Bach1* constitutively represses *Spic* expression in monocytes (Haldar et al., 2014). *Bach1* deficiency, similar to LPS treatment, promoted *Spic* expression in Ly6C^{lo}TremL4^{hi} patrolling monocytes (Figure 1D). Therefore, the two major subsets of circulating monocytes differ in their ability to express *Spic*.

Monocytes can differentiate into macrophages or dendritic cells (DCs), and we previously showed that TREML4 expression marks the loss of DC differentiation potential in circulating monocytes (Briseño et al., 2016). The selective expression of *Spic* in TremL4+ monocytes suggests that the capacity to express *Spic* is linked to macrophage identity. To further test this, we obtained *Zbtb46*^{GFP} reporter mice in which GFP expression is restricted to DCs (Satpathy et al., 2012). We generated a mixed population of macrophages and DCs *in vitro* by culturing *Zbtb46*^{GFP} bone marrow cells with granulocyte-macrophage colony-stimulating factor (GM-CSF) (Helft et al., 2015). LPS treatment in this setting strongly induced *Spic* in ZBTB46-GFP^{-}F4/80^{hi} macrophages but not ZBTB46-GFP^{+}F4/80^{-} DCs, confirming the specificity of *Spic* to the macrophage lineage (Figure 1E).

We next examined whether tissue macrophages induce *Spic* during inflammation. High levels of heme can induce *Spic* in iron-recycling macrophages of the spleen, bone marrow, and liver (Haldar et al., 2014). Hence, we excluded these organs from our initial analyses to disentangle the impact of heme from TLR activation on *Spic*. Intraperitoneal LPS induced *Spic* in monocytes and macrophages of various tissues but not in DCs (Figures 2A and S1A). Macrophages in the intestinal tract are exposed to TLR ligands derived from gut microbiota (Bain et al., 2013). Correspondingly, we detected *Spic* in macrophages of the large intestine at steady state (Figure. 2B). Gut macrophages are further divided into three major subsets: (1) CD4+Tim4+ subset that is maintained by local proliferation, (2) CD4+Tim4− subset with slow turnover from monocytes, and (3) CD4−Tim4− subset with rapid turnover from circulating monocytes (Shaw et al., 2018). We detected *Spic* in all three subsets (Figure 2B). Our findings are consistent with a recent report that also cited *Spic* expression in gut macrophages (Kayama et al., 2018). Notably, the level of *Spic* was higher in the colon than in the small intestine, which likely reflects the higher density of microbiota in the colon (Figure 2C). Correspondingly, *Spic* expression was lower in the colon of germ-free than in conventionally housed mice (Figure 2D).

TLR activation promotes monocyte differentiation into macrophages (Krutzik et al., 2005). Hence, TLR-induced *Spic* in tissues may represent macrophages newly differentiated

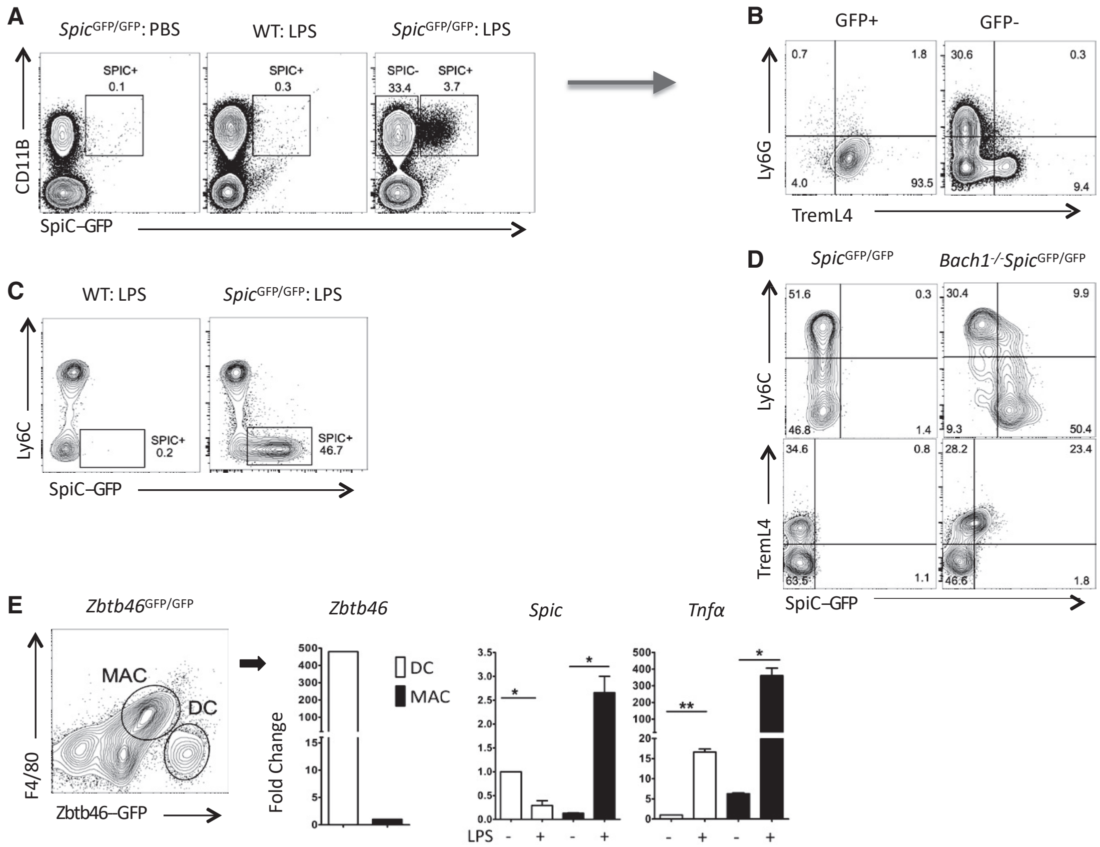

Figure 1. TLR Activation Induces *Spic* in Monocytes and Macrophages

(A–C) LPS (50 μg) or PBS (control) was injected intraperitoneally (i.p.) into mice of indicated genotypes (headers). Peripheral blood was collected 3 days later. Shown are the flow cytometry plots (FCSs) with indicated markers. (A) Cells pre-gated for CD45+ singlets expresses SPIC (GFP+) selectively in CD11B+ myeloid cells. (B) Expression of indicated markers on cells gated on SPIC expression (A, arrow). (C) FCS showing SPIC expression in monocyte subsets defined by Ly6C (pre-gated for CD45+ singlets).

(D) FCS of circulating monocytes (CD45+LY6G−CD115+) from mice of indicated genotypes (header) showing *Spic* expression in circulating leukocytes.

(E) *Zbtb46*$^{GFP/GFP}$ bone marrow cells were cultured in GM-CSF for 7 days. F4/80^{hi}Zbtb46GFP^{-} macrophages (MAC) and F4/80^{lo}Zbtb46GFP^{+} DCs were purified by fluorescence-assisted cell sorting (FACS), cultured in media without cytokines, and treated with LPS. Cells were harvested 24 h later, and qRT-PCR was performed for indicated genes (y axis, normalized to *18S rRNA*). MACs but not DCs induced *Spic* while both cell types increased *Tnfα* expression with LPS.

FCS, numbers represent percentage of cells within indicated gate. (A–D) Represents ≥3 experiments with ≥3 mice per group. qRT-PCR, data representative of ≥3 independent experiments; and graphs show a single experiment with n ≥2 per group. Results are expressed as mean ± SEM. p ≤ 0.05 (*), p ≤ 0.01 (**), p ≤ 0.001 (***), and p ≤ 0.0001 (****). See also Figure S1.

from infiltrating monocytes, or they may represent pre-existing tissue-resident macrophages. To test whether tissue-resident macrophages can induce *Spic*, we purified lung and peritoneal resident macrophages and exposed them to LPS *in vitro*, finding robust *Spic* induction (Figures 2E and 2F). Next, we asked whether macrophages that already express high levels of *Spic* at the steady state (splenic red pulp macrophages [RPMs] and liver Kupffer cells) can further induce it upon TLR activation. We isolated *Spic*-high and *Spic*-negative macrophages from spleen and liver of Spic$^{GFP/GFP}$ mice and exposed them to LPS *ex vivo*. Although LPS further increased *Spic* expression in *Spic*-high macrophages, the level of induction was significantly less (~2× versus >10×) than that in macrophages that did not express *Spic* prior to LPS exposure (Figures 2G, 2H, and S1B). Hence, TLR-induced *Spic* is a conserved feature of macrophages.

Spic Downregulates Inflammatory Responses in Activated Macrophages

The spleen contains *Spic*-high RPMs and their *Spic*-low precursors (PreRPM) (Haldar et al., 2014). *Spic*$^{-/-}$ mice lack RPM but not PreRPMs (Figure 3A). A microarray-based gene expression comparison of wild-type (WT) and *Spic*$^{-/-}$ PreRPM revealed a prominent inflammatory signature in the latter, suggesting an anti-inflammatory function of *Spic* (Figure 3B). Indeed, a recent study suggested that heme-induced *Spic* downregulates inflammation in a murine model of dextran sodium sulfate(DSS)-induced colitis (Kayama et al., 2018). Therefore, we further examined whether TLR-induced *Spic* serves an anti-inflammatory function. We treated *Spic*$^{GFP/GFP}$ bone-marrow-derived macrophages (BMDMs) with LPS *in vitro* and isolated SPIC+ and SPIC− macrophages. In this setting, SPIC+ macrophages expressed lower pro- and higher anti-inflammatory cytokines

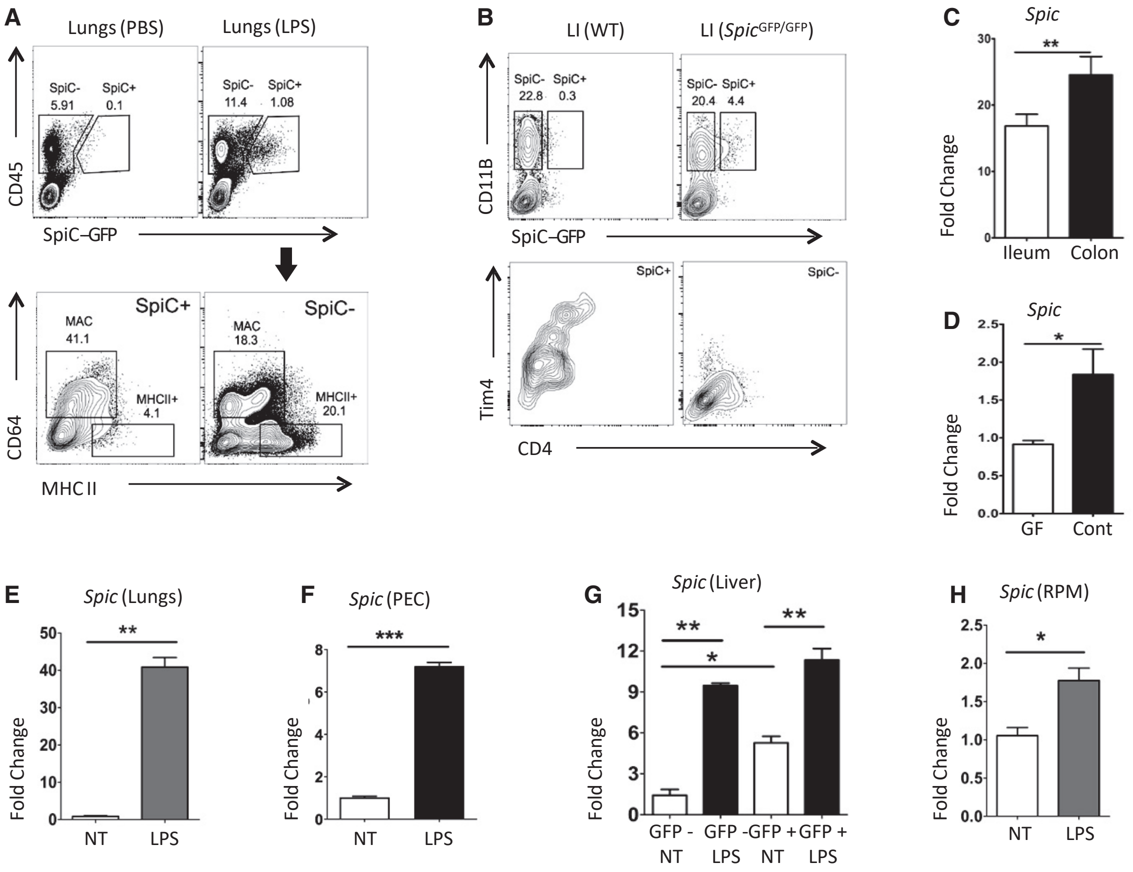

Figure 2. TLR Activation Induces *Spic* in the Tissue Macrophages

(A) *Spic*$^{GFP/GFP}$ mice were treated with i.p. LPS (75 μg in PBS) or control (PBS), and lungs were harvested 48 h after treatment. Top: FCS with indicated markers on singlets, highlighting SPIC expression. Bottom: distribution of indicated markers on SPIC+ and SPIC− cells (gating shown in top panels, arrow).

(B) FCS with indicated markers in CD45+ live (7AAD−) singlets from large intestine of *Spic*$^{GFP/GFP}$ and wild-type (WT) mice, showing Tim4 and CD4 expression largely restricted to SPIC+ cells.

(C) Published gene expression profiles (microarray based) of murine ileum and colon were downloaded from a public database (GEO: GSE32513). Shown are expression values (linear scale) of *Spic*.

(D) qRT-PCR-based expression of *Spic* (normalized to *Hprt*) in colon obtained from conventionally raised (Cont) and germ-free (GF) mice.

(E) Lung macrophages (CD45+CD64+CD11C+) were isolated by FACS, cultured with M-CSF for 12 h, and treated with LPS (1 μg/ml). RNA was extracted 10 h after LPS treatment, and the expression (normalized to *Hprt*) of *Spic* was measured by qRT-PCR.

(F) Peritoneal cells (PECs) were cultured with M-CSF for 12 h, followed by LPS (1 μg/ml) treatment. RNA was extracted 20 h after treatment, and the expression (relative to *Hprt*) of *Spic* was measured by qRT-PCR.

(G) CD45+F4/80+GFP+ and CD45+F4/80+GFP− liver macrophages were purified by FACS, cultured with M-CSF for 12 h, and treated with LPS (1 μg/ml). RNA was extracted 14 h after LPS treatment, and the expression (normalized to *18S rRNA*) of *Spic* was measured by qRT-PCR.

(H) RPMs from *Spic*$^{GFP/GFP}$ spleen were purified by FACS, cultured with M-CSF for 12 h, and treated with LPS (1 μg/ml). RNA was extracted 16 h after LPS treatment, and the expression (normalized to *Hprt*) of *Spic* was measured by qRT-PCR.

FCS, numbers represent percentage of cells within indicated gate. (A and B) Represent $\geq$3 experiments with $\geq$3 mice per group. qRT-PCR, data representative of $\geq$ 3 independent experiments; and graphs show a single experiment with n $\geq$2 per group. Results expressed as mean ± SEM. $p \leq 0.05$ (*), $p \leq 0.01$ (**), $p \leq 0.001$ (***), and $p \leq 0.0001$ (****). See also Figures S1, S6, and S7.

(Figures 3C). These differences were maintained when SPIC+ and SPIC− macrophages were re-exposed to LPS (Figure S2A). Hence, high levels of *Spic* expression marks macrophages with lower inflammatory responses. Next, we compared LPS responses of WT and *Spic*$^{-/-}$ BMDMs. Consistent with the above observations, *Spic* deficiency engendered higher pro-inflammatory cytokine expression (Figures 3D and 3E). Correspondingly, *Spic*$^{-/-}$ mice showed higher body temperature, higher levels of circulating tumor necrosis factor α (TNF-α), and increased lung *Nos2* than WT mice upon intraperitoneal LPS exposure (Figures 3F and S2B).

Bach1$^{-/-}$ macrophages have been shown to display an anti-inflammatory phenotype (Harusato et al., 2013). Because *Bach1*$^{-/-}$ macrophages also express high levels of *Spic*, we asked whether *Spic* might drive anti-inflammatory properties of *Bach1*$^{-/-}$ macrophages. We generated *Bach1*$^{-/-}$: *Spic*$^{-/-}$ (double knockout [DKO]) mice and compared inflammatory responses of DKO and *Bach1*$^{-/-}$ BMDMs. In the setting of LPS exposure, we found

CellPress
OPEN ACCESS

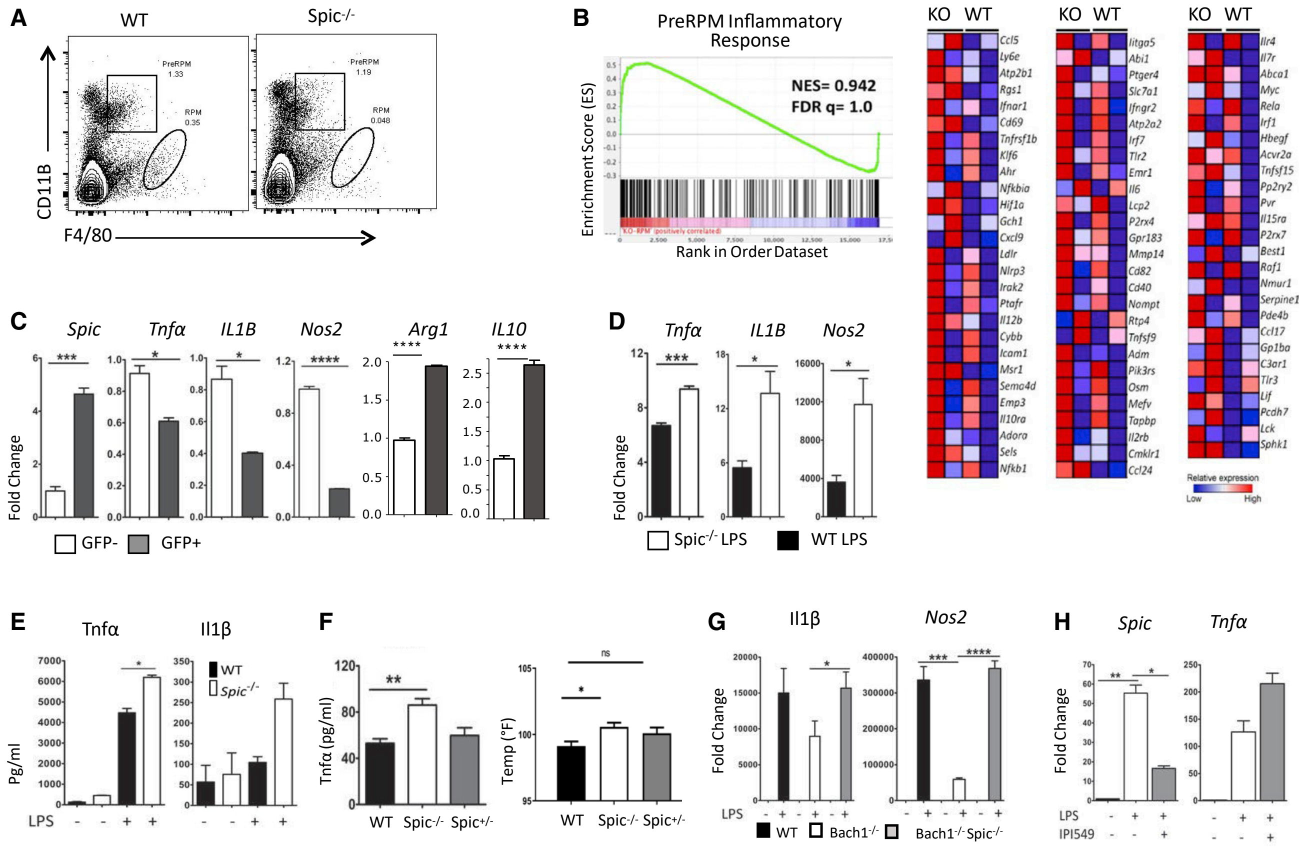

Figure 3. *Spic* Controls Inflammatory Response in Macrophages

(A) FCS (pre-gated on singlets) on splenocytes from WT and *Spic*$^{-/-}$ mice show drastically reduced RPMs but relatively normal PreRPMs in *Spic*-deficient spleen.

(B) PreRPM from WT and *Spic*$^{-/-}$ spleen (two mice per genotype) were purified by FACS and subjected to microarray-based gene expression profiling (Affymetrix, mouse gene_2.0ST). Shown are the gene set enrichment analyses (GSEAs) for the signature associated with inflammatory response (left) and the corresponding heatmap based on differentially expressed genes (nominal p = 0.504) between the two genotypes (right).

(C) BMDMs from *Spic*$^{GFP/GFP}$ mice were treated with LPS (1 μg/ml). After 48 h, GFP+ (SPIC-expressing) and GFP− (SPIC−) cells were purified by FACS and RNA extracted, and the expression (normalized to *Hprt*) of indicated genes (y axis, relative to GFP− cells) was measured by qRT-PCR.

(D) Mo-MACs from WT and *Spic*$^{-/-}$ mice were treated with LPS (1 μg/ml), RNA was extracted 24 h later, and the expression (normalized to *Hprt*) of indicated genes (y axis, relative to WT no treatment) was measured by qRT-PCR.

(E) BMDMs from WT and *Spic*$^{-/-}$ mice were treated with LPS (1 μg/ml). After 16 h, the amount (y axis) of indicated cytokines released into the media was measured by ELISA.

(F) WT, *Spic*$^{-/-}$, and *Spic*$^{+/-}$ mice were treated (i.p.) with LPS (7.5 μg/gm). Rectal temperature (right graph) and plasma TNF-α (ELISA, left graph) were measured 24 h after treatment.

(G) BMDMs from WT, *Bach1*$^{-/-}$, and *Bach1*$^{-/-}$: *Spic*$^{-/-}$ double knockout (DKO) were treated with LPS (1 μg/ml). RNA was extracted 24 h later, and the expression (normalized to *Hprt*) of indicated genes was measured (y axis, relative to WT non-treated group) by qRT-PCR.

(H) Mo-MACs from WT mice were treated with LPS (1 μg/ml) with or without PI3Kγ inhibitor, IPI549 (100 nM). RNA was extracted 20 h later, and the expression (normalized to *Hprt*) of indicated genes was measured (y axis, relative to no treatment group) by qRT-PCR.

FCS, numbers represent percentage of cells within indicated gate. (A) Represents ≥3 experiments with ≥3 mice per group. qRT-PCR, data representative of ≥3 independent experiments; and graphs show single experiment with n ≥2 per group. Results expressed as mean ± SEM. $p \leq 0.05$ (*), $p \leq 0.01$ (**), $p \leq 0.001$ (***), and $p \leq 0.0001$ (****). See also Figure S2.

that the loss of *Spic* reversed the anti-inflammatory phenotype of *Bach1*$^{-/-}$ BMDMs (Figure 3G). Finally, a recent study showed that phosphatidylinositol 3 kinase (PI3K)-γ signaling promotes a "switch" from a pro- to anti-inflammatory phenotype in activated macrophages (Kaneda et al., 2016). Inhibiting PI3K-γ in TLR-activated macrophages reduced *Spic* and increased pro-inflammatory cytokine expression (Figure 3H). Taken together, these findings suggest that *Spic* downregulates the transcription of pro-inflammatory cytokines in activated macrophages.

Spic Promotes Iron Export in Activated Macrophages

To identify the genetic targets of *Spic* in inflammatory settings, we compared the gene expression profile (microarray based) of patrolling monocytes from *Spic*$^{+/-}$ and *Spic*$^{-/-}$ mice treated with intraperitoneal LPS. Gene set enrichment analysis (GSEA) of differentially expressed genes revealed a hallmark for heme metabolism in *Spic*$^{-/-}$ monocytes, which is typically associated with cells containing high levels of heme and iron (Figure 4A). *Fpn* is the only known mammalian exporter of iron

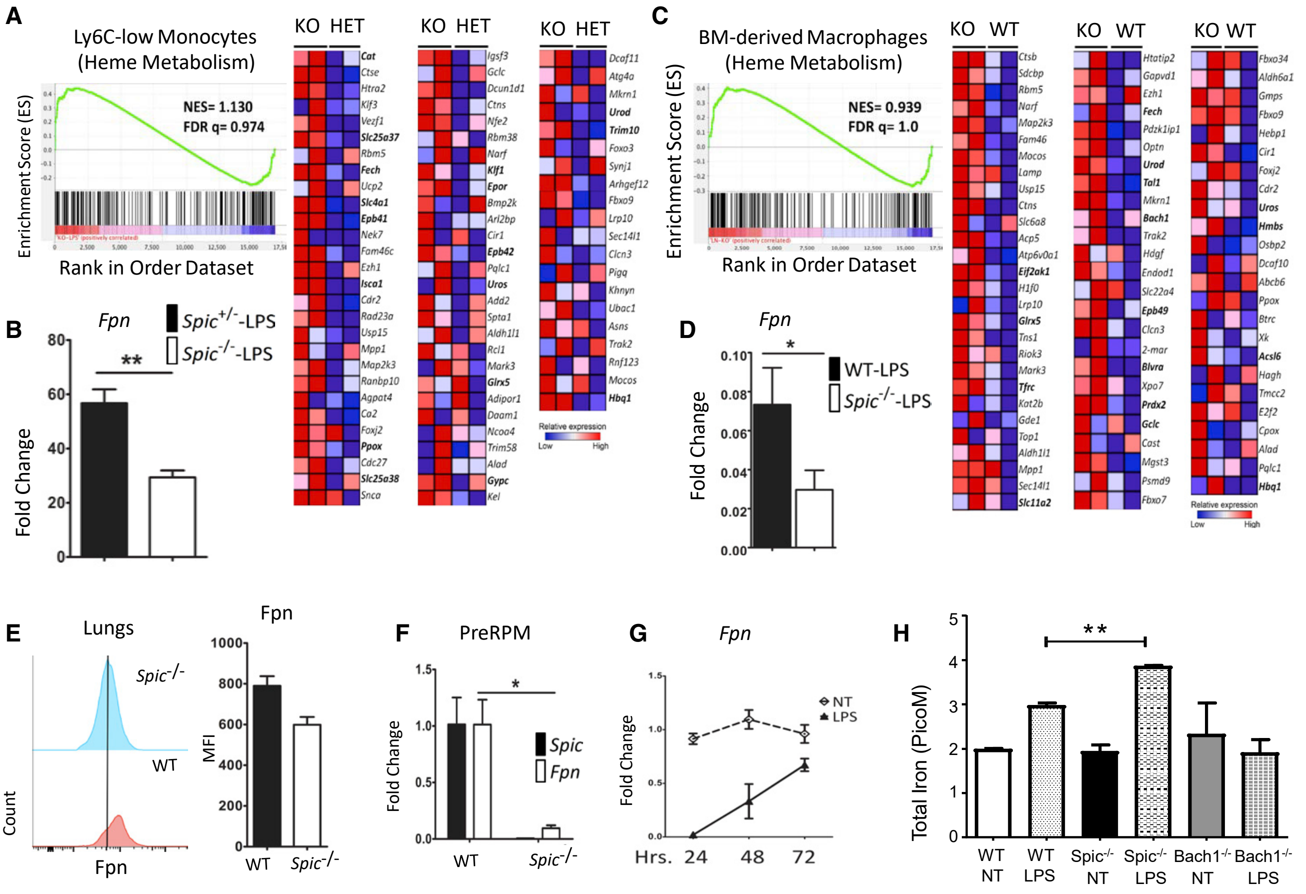

Figure 4. *Spic* Regulates Ferroportin-Mediated Iron Export in Macrophages

(A) *Spic*$^{+/-}$ and *Spic*$^{-/-}$ mice (three mice per group) were treated with LPS (30 µg/mouse). Patrolling monocytes (CD45+CD11B+CD115+LY6C−) were purified 72 h after treatment and subjected to microarray-based (affymetrix, mouse gene 2.0_ST) gene expression profiling. Shown are the GSEA plots for the hallmark of heme metabolism (left) and corresponding heatmap based on differentially expressed genes (nominal $p < 0.01$) between the two genotypes (right).

(B) Expression (y axis, linear scale) of *Fpn* from the microarray data.

(C) WT and *Spic*$^{-/-}$ BMDMs (two mice per group) were treated with LPS (100 ng/ml), RNA was extracted 24 h later, and microarray-based (affymetrix, mouse gene 2.0_ST) gene expression profiling was performed. Shown are the GSEA plots for the hallmark of heme metabolism (left) and the corresponding heatmap based on differentially expressed genes (nominal $p = 0.457$) between the two genotypes (right).

(D) Mo-MACs from WT and *Spic*$^{-/-}$ mice were treated with LPS (1 µg/ml), RNA was extracted 16 h ater, and the expression (normalized to *18S rRNA*) of *Fpn* was measured (y axis relative to a non-treated group) by qRT-PCR.

(E) WT or *Spic*$^{-/-}$ mice were treated (i.p.) with LPS (150 µg/mouse) twice 48 h apart. Lungs were harvested 48 h after the final LPS treatment, and levels of FPN protein measured by flow cytometry (left). The bar graph (right) shows quantification of mean fluorescent intensity (MFI) of FPN staining.

(F) PreRPM (CD11B^{hi}F4/80^{lo}) from WT and *Spic*$^{-/-}$ spleen were purified by FACS, RNA was extracted, and the levels (normalized to *18S rRNA*) of *Spic* and *Fpn* were measured (y axis, relative a WT PreRPM) by qRT-PCR.

(G) BMDMs from WT mice were treated with LPS (100 ng/ml), RNA was extracted at indicated time points (x axis), and the levels (normalized to *18S rRNA*) of *Fpn* were measured (y axis, relative to non-treated group) by qRT-PCR.

(H) BMDMs of indicated genotypes were cultured for 7 days, after which the medium was removed, and fresh medium containing 100 mM of FeSO4 was added, followed by treatment with LPS (1 µg/mL) or PBS (control). Intracellular iron was measured 24 h later. Graph is representative of five independent experiments. The replicates for each individual experiment are technical replicates for the assay.

qRT-PCR, data representative of ≥3 independent experiments. Plots show a single experiment with n ≥2 per group. Results are expressed as mean ± SEM. $p \le 0.05$ (*), $p \le 0.01$ (**), $p \le 0.001$ (***), and $p \le 0.0001$ (****). See also Figures S2 and S3.

and, remarkably, was one of the most downregulated genes in LPS-exposed *Spic*$^{-/-}$ monocytes (Figure 4B). This was independently validated by measuring *Fpn* expression in classical and patrolling monocytes from mice treated with LPS (Figure S2C). We found a similar trend *in vitro*, where LPS-treated *Spic*$^{-/-}$ macrophages expressed higher levels of genes involved in heme metabolism (Figure 4C) and lower levels of *Fpn* (Figure 4D). Correspondingly, lung macrophages and cells from the peritoneal cavity of *Spic*$^{-/-}$ mice showed lower FPN protein expression after LPS exposure *in vivo* (Figures 4E and S2D). Finally, splenic PreRPM expressed lower *Fpn* than their WT counterpart (Figure 4F). These findings suggest that *Spic*

promotes *Fpn* expression and are consistent with previous observations of higher splenic iron in *Spic*$^{-/-}$ mice (Kohyama et al., 2009).

LPS strongly downregulates *Fpn* transcription in macrophages to sequester iron during inflammation (Abreu et al., 2018). Its expression gradually recovers during the resolution of inflammation, presumably to facilitate an efflux of the sequestered iron (Figure 4G). Our findings suggest that the "recovery" of *Fpn* expression in activated macrophages is regulated by *Spic*. In contrast, the transcription of ferritin heavy and light chains, which are key players in intracellular iron storage, did not show significant alterations with *Spic* deficiency (Figure S2E). Hence, *Spic* may selectively impact iron efflux by FPN without directly affecting other elements of cellular iron homeostasis.

Most studies of macrophage iron sequestration during inflammation have focused on the paracrine circuit involving Hepcidin (*Hamp*), an inflammation-induced hormone produced by hepatocytes that mediates the degradation of surface FPN on macrophages (Ganz and Nemeth, 2015). Our findings show a cell-intrinsic transcriptional circuitry that may regulate macrophage iron efflux during the resolution of inflammation. This is reminiscent of RPM, suggesting that TLR activation induces an RPM-like phenotype in macrophages. Indeed, a recent study showed that chronic TLR7/9 signaling induces an RPM-like macrophage differentiation from circulating monocytes (Akilesh et al., 2019). We found that *Spic* deficiency is associated with a trend toward higher liver *Hamp* upon TLR exposure (Figure S2F). Therefore, the impact of *Spic* on FPN-mediated iron regulation is 2-fold: (1) regulation of *Fpn* transcription within macrophages and (2) regulation of FPN protein stability by *Hamp*. However, *Hamp* expression within macrophages itself did not change significantly with *Spic* deficiency, suggesting that higher *Hamp* in the liver likely reflects a higher production by hepatocytes in response to higher inflammation in *Spic*$^{-/-}$ mice (Figure S2G).

Spic$^{-/-}$ macrophages upregulate *Fpn* in response to heme, much like their WT counterparts (Haldar et al., 2014). Hence, *Spic* is not required for *Fpn* expression, instead promoting *Fpn* transcription in specific contexts, such as PAMP-activated macrophages. This raises the question of what impact, if any, does *Spic*-regulated *Fpn* have on macrophage iron storage during inflammation. To address this, we devised an *in vitro* assay where WT, *Bach1*$^{-/-}$, and *Spic*$^{-/-}$ BMDMs were loaded with iron (ferrous sulfate) prior to LPS exposure, followed by measurement of total intracellular iron. As expected, LPS led to increased intracellular iron in WT and *Spic*$^{-/-}$ macrophages (Figure 4H). *Bach1* is a negative regulator of *Fpn*, and *Bach1*$^{-/-}$ macrophages express high levels of *Fpn*. Correspondingly, *Bach1*$^{-/-}$ macrophages did not show significant increases in intracellular iron with LPS (Figure 4H). Importantly, LPS-exposed *Spic*$^{-/-}$ macrophages displayed higher intracellular iron than their WT counterparts (Figure 4H). Taken together, these findings show that transcriptional fine-tuning of *Fpn* expression by *Spic* regulates macrophage iron storage during inflammation.

The dramatic downregulation of *Fpn* with LPS appears to be an important driver of iron accumulation in activated macrophages. Nonetheless, the underlying molecular mechanism is unclear. *Bach1* is a negative regulator of *Fpn* (Igarashi and Watanabe-Matsui, 2014). However, LPS downregulated *Fpn* in *Bach1*$^{-/-}$ macrophages to the same extent as in the WT (Figure S3A). The transcription factor *Nrf2* is known to promote *Fpn* and heme oxygenase 1 (*Ho1*) expression (Ma, 2013). LPS did not significantly alter *Nrf2* expression, and *Ho1* levels increased with LPS, ruling out *Nrf2* transcriptional downregulation as a mediator of LPS-induced suppression of *Fpn* (Figures S3B and S3C). Blocking new protein synthesis with cycloheximide did not affect *Fpn* downregulation by LPS, indicating the role of a preformed factor (Figure S3D). Activation of preformed components of the NF-κB pathway play a critical role in LPS signaling, but both pharmacological inhibition and genetic disruption of NF-κB signaling failed to block *Fpn* downregulation (Figures S3E and S3F). Hence, LPS-induced activation of a preformed factor likely mediates *Fpn* downregulation, and *Spic* may facilitate *Fpn* recovery by suppressing this "unidentified factor." This idea is also consistent with previous observations that *Spic* generally acts as a transcriptional repressor.

TLR Activation Induces *Spic* by a Heme-Independent and NF-κB-Dependent Mechanism

Heme can induce *Spic* by proteasome-dependent degradation of BACH1 (Haldar et al., 2014; Kayama et al., 2018). Therefore, hemophagocytosis or heme accumulation by activated macrophages may explain TLR-induced *Spic*. However, *in-vitro*-cultured BMDMs treated with TLR agonists strongly induced *Spic*, suggesting a heme-independent mechanism (Figure 5A). LPS treatment of *Bach1*$^{-/-}$ BMDMs (which constitutively express *Spic*) further increased *Spic*, supporting a BACH1-independent pathway for *Spic* induction (Figure 5B). LPS induced *Spic* at a later time point than heme (Figure 5C), and LPS treatment did not reduce *Bach1* transcript levels in macrophages (Figure 5D). These results suggest that TLR activation induces *Spic* by a mechanism distinct from the previously described heme and BACH1-dependent pathway.

TLR4 activates several latent transcription factors, including NF-κB and AP-1. Although AP-1 inhibition did not affect *Spic* expression, blocking NF-κB by bot 64, a small molecule inhibitor of the inhibitor of NF-κB kinase beta (Iκκ-2), abrogated *Spic* induction by LPS *in vitro* and reduced it *in vivo* (Figures 5E, 5F, and S4A). *Rel* knockout BMDMs also showed reduced *Spic* induction with LPS, further confirming a role of NF-κB (Figure 5G). Notably, NF-κB blockade also reduced *Spic* in *Bach1*-deficient macrophages, suggesting a central role for NF-κB in *Spic* regulation (Figure 5H). Correspondingly, heme-mediated *Spic* expression also showed dependence on NF-κB activity (Figure S4B). NF-κB may directly promote *Spic* transcription (primary response gene) or indirectly by transcribing another factor (secondary response gene). To address this, we blocked new protein synthesis by treatment with cycloheximide, which completely blocked *Spic* induction by LPS (Figure 5I). Taken together, these results show that *Spic* is a TLR-induced and NF-κB-dependent secondary response gene in activated macrophages.

IFNγ Signaling Suppresses *Spic* Expression

The aforementioned findings show that *Spic* downregulates inflammatory responses and promotes iron-efflux in macrophages. Although this is beneficial during the resolution of

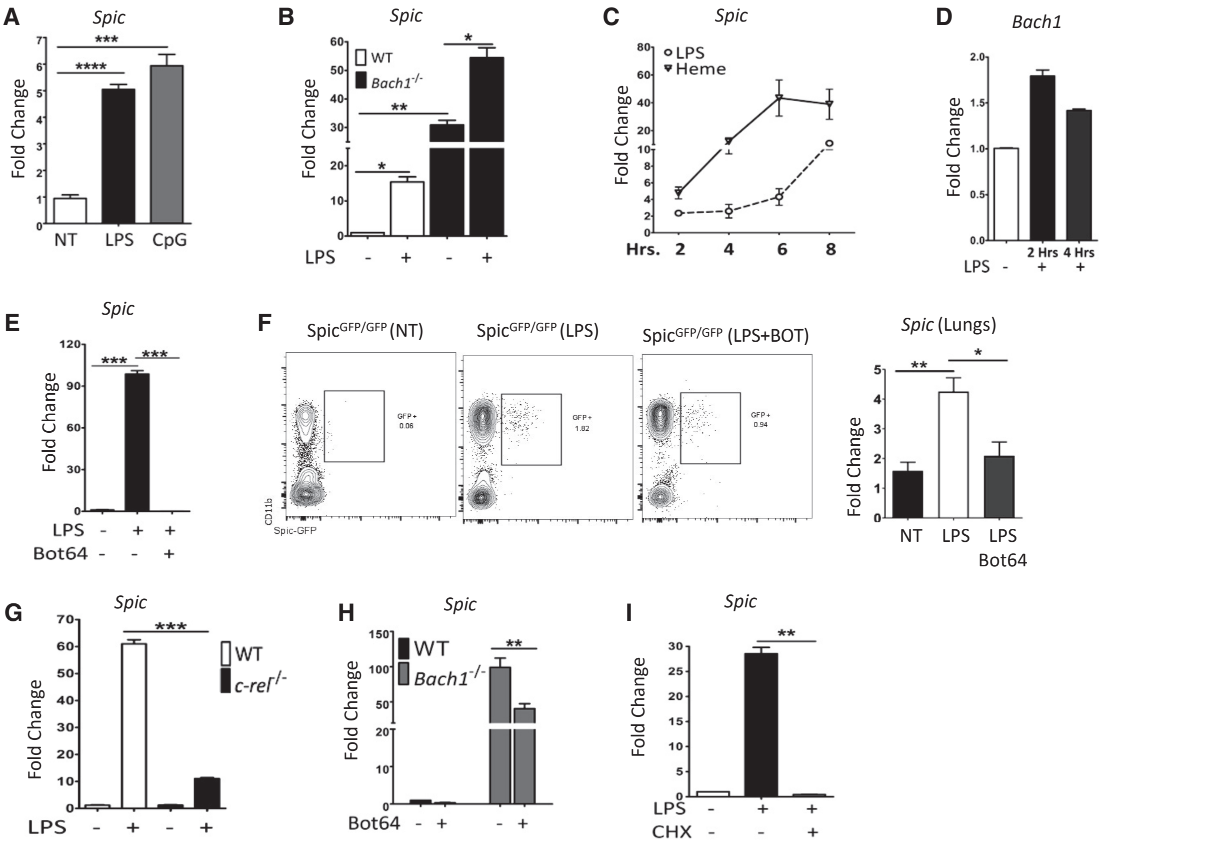

Figure 5. NF-κB Is Required for *Spic* Expression

(A) BMDMs from WT mice were treated with LPS (100 ng/ml) or TLR9 ligand CpG (30 μg/ml), RNA was extracted 8 h later, and the expression (normalized to *18S rRNA*) of *Spic* was measured (y axis, relative to no treatment) by qRT-PCR.

(B) WT and *Bach1*$^{-/-}$ Mo-MACs were treated with LPS (100 ng/ml), RNA was harvested 24 h later, and the expression (normalized to *Hprt*) of *Spic* was measured (y axis, relative to WT no treatment) by qRT-PCR.

(C) WT BMDMs were treated with LPS (100 ng/ml) or hemin (80 μM), RNA was extracted at indicated (x axis, hours) time points, and the expression (normalized to *18S rRNA*) of *Spic* was measured (y axis, relative to no treatment) by qRT-PCR.

(D) WT Mo-MACs were treated with LPS (1 μg/ml), RNA was extracted at indicated time points (x axis), and expression of *Bach1* (normalized to *18S rRNA*) was measured (y axis, relative to no treatment) by qRT-PCR.

(E) WT Mo-MACs were treated with LPS (1 μg/ml) with or without an Iκκ-2 inhibitor, Bot64 (10 μM). RNA was extracted 18 h later, and the expression (normalized to *18S rRNA*) of *Spic* was measured (y axis, relative to no treatment) by qRT-PCR.

(F) Mice of indicated genotypes (header) were treated with LPS with or without Bot64 (i.p.). Bot64 treatment (60 mg/kg/day for 3 days) started 24 h before LPS (single dose, 100 μg/mouse) treatment. Peripheral blood and lungs were collected 24 h after LPS treatment. FCS plots (left) show the distribution of indicated markers in peripheral blood (cells pre-gated for CD45+ Ly6G− singlets). The expression (relative to *Hprt*) of *Spic* in the lungs (measured by qRT-PCR) is shown in the right plot.

(G) WT or *c-rel*$^{-/-}$ Mo-MACs were treated with LPS (1 μg/ml), RNA was extracted 16 h later, and the expression (normalized to *Hprt*) of *Spic* was measured (y axis, relative to WT no treatment) by qRT-PCR.

(H) WT and *Bach1*$^{-/-}$ Mo-MACs were treated with an Iκκ-2 inhibitor, Bot64 (10 μM), RNA was extracted 14 h later, and the expression of *Spic* (normalized to *18S rRNA*) was measured (y axis, relative to WT no treatment) by qRT-PCR.

(I) WT Mo-MACs were treated with LPS (1 μg/ml) alone or with cycloheximide (10 μg/ml, added 1 h before LPS) to inhibit new protein synthesis. RNA was harvested 8 h later, and the expression (normalized to *Hprt*) of *Spic* was measured (y axis, relative to no treatment) by qRT-PCR.

FCS, numbers represent percentage of cells within indicated gate. (F) Represents 2 experiments with ≥3 mice per group. qRT-PCR, representative of ≥3 independent experiments and graphs show single experiment with n ≥2 per group. Results expressed as mean ± SEM. $p \leq 0.05$ (*), $p \leq 0.01$ (**), $p \leq 0.001$ (***), and $p \leq 0.0001$ (****). See also Figure S4.

inflammation, it is detrimental to host defense against pathogens. Infection that activates macrophages is unlikely to be fully resolved within the period of *Spic* induction (6–8 h after TLR activation; Figure 5C). Hence, induction of *Spic* in the setting of a true infection appears counter-intuitive, and we wondered whether there are additional constraints on *Spic* expression.

Local IFN levels are elevated during infection. Therefore, we examined whether the presence of IFNs affect *Spic* expression. Remarkably, IFNγ strongly inhibited LPS-mediated *Spic* expression in BMDMs, which was dependent on STAT1 activity (Figure 6A). Type-1 IFNs also showed a similar trend, albeit to a much lesser extent (Figure S5A). Correspondingly, pretreatment

CellPress
OPEN ACCESS

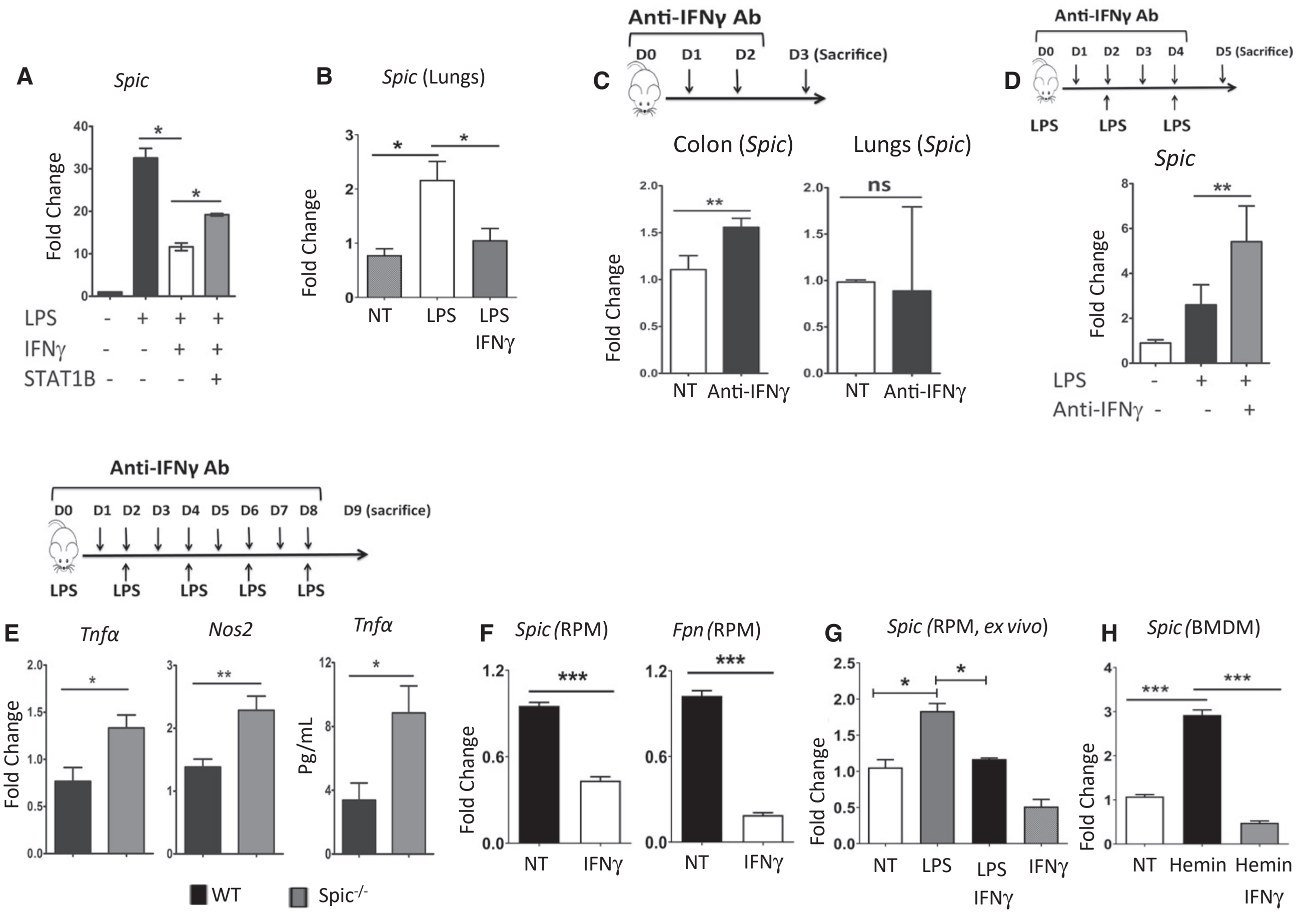

Figure 6. IFNγ Suppresses *Spic* Expression

(A) WT BMDMs were treated with LPS (1 μg/ml), IFNγ (50 ng/ml), or STAT1 blocker fludarabine (50 μM). RNA was extracted 20 h later, and the expression (normalized to *Hprt*) of *Spic* was measured (y axis, compared to no treatment group) by qRT-PCR.

(B) Mice were treated (i.p.) with LPS (once, 100 μg/mouse) with or without recombinant IFNγ (20 μg/mouse per dose for a total of five doses). IFNγ treatment was started 1 day before LPS injection. Lungs were harvested within 24 h of LPS treatment, RNA extracted, and the expression of *Spic* was measured (relative to *Hprt*) by qRT-PCR. Five mice per treatment group.

(C) Anti-IFNγ antibody (200 μg/mouse) was injected i.p. every 24 h for 3 days. At 24 h after the last treatment, mice were euthanized, and indicated organs were harvested. RNA was extracted, and the levels (normalized to *Hprt*) of *Spic* (y axis, relative to a non-treated mouse) were measured by qRT-PCR.

(D) WT mice were treated (i.p.) with LPS (150 μg/mouse, three treatments 48 h apart) with or without anti-IFNγ antibody (200 μg/mouse, daily, starting with LPS treatment). Lungs were harvested 24 h after the last LPS treatment, RNA was extracted, and the expression (normalized to *Hprt*) of *Spic* (y axis, relative to non-treated mice) was measured by qRT-PCR.

(E) WT and *Spic*$^{-/-}$ mice were treated (i.p.) with LPS (150 μg/mouse, 5 doses, 48 h apart) with or without anti-IFNγ antibody (200 μg/mouse, daily, starting with LPS treatment). Lungs were harvested 24 h after the final LPS treatment, RNA was extracted, and the levels (normalized to *Hprt*) of the indicated genes (y axis, relative to non-treated mice) were measured using qRT-PCR (first two graphs). TNF-α levels were also measured using ELISA (final graph) in plasma from blood collected 24 h before sacrificing the mice.

(F) *Spic*$^{GFP/GFP}$ mice were treated with recombinant IFNγ (20 μg/mouse per dose, every 12 h, for 3 days), followed by purification of splenic RPMs by FACS (using GFP expression). RPMs from untreated *Spic*$^{GFP/GFP}$ mice served as controls. RNA was extracted, and the expression (relative to *Hprt*) of indicated genes was measured by qRT-PCR.

(G) RPMs purified from *Spic*$^{GFP/GFP}$ splenocytes by FACS were cultured with macrophage colony stimulating factor (M-CSF). After 6 h, cells were treated with vehicle, LPS (1μg/ml), IFNγ (50ng/ml), or IFNγ + LPS. 16 h after treatment, RNA was extracted, and the expression (relative to *Hprt*) of *Spic* was measured by qRT-PCR.

(H) WT BMDMs were treated with heme (80 μM) with or without IFNγ (50 ng/ml). 4 h after treatment, RNA was extracted, and expression (relative to *Hprt*) of *Spic* was measured by qRT-PCR.

qRT-PCR, data representative of ≥3 independent experiments. Plots show a single experiment with n ≥2 per group. Results expressed as mean ± SEM. $p \leq 0.05$ (*), $p \leq 0.01$ (**), $p \leq 0.001$ (***), and $p \leq 0.0001$ (****). See also Figure S5.

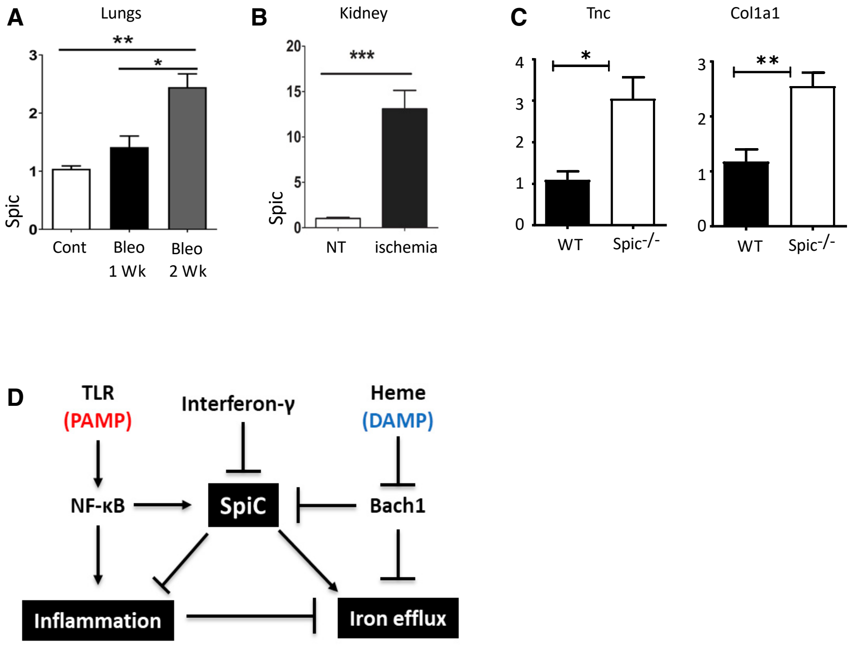

Figure 7. *Spic* Expression Is Induced in Sterile Inflammation

(A) WT (C57BL/6J) mice were treated (intratracheal [i.t.]) with bleomycin sulfate (3 U/kg) or water (control). Mice were euthanized 1 or 2 weeks after treatment, RNA was extracted from lungs, and the expression (relative to *Hprt*) of *Spic* was measured using qRT-PCR.

(B) Kidneys were harvested from control or 30 days after inducing renal ischemia in WT (C56BL/6J) mice, RNA was extracted, and the expression (relative to *Hprt*) of *Spic* was measured by qRT-PCR. Experiment representative of two experiments with n $\geq$3 mice per group per experiment.

(C) Mice of indicated genotypes were treated (i.t.) with bleomycin sulfate (3 U/kg) and were euthanized 3 weeks later, lungs were harvested, and the expression (normalized to *Hprt*) of indicated genes was measured by qRT-PCR. Data are representative of four independent experiments with $\geq$ 2 mice per genotype/condition.

(D) Representative model for the regulation and function of *Spic*. PAMP, pathogen-associated molecular pattern; DAMP, damage-associated molecular pattern.

qRT-PCR, data representative of $\geq$2 independent experiments. Plots show a single experiment with n $\geq$2 per group. Results are expressed as mean ± SEM. $p \leq 0.05$ (*), $p \leq 0.01$ (**), $p \leq 0.001$ (***), and $p \leq 0.0001$ (****). See also Figures S6 and S7.

of mice with IFNγ prior to LPS exposure suppressed *Spic* induction *in vivo* (Figures 6B and S5B).

We showed above that gut macrophages express *Spic* in response to local microbiota. Consistent with the suppressive effects of IFNγ, treatment with an anti-IFNγ antibody led to increased *Spic* in colonic macrophages but not lungs, an organ we used as a control due to lower exposure to TLR ligands at steady state than that in the gut (Figure 6C). Furthermore, IFNγ blockade augmented LPS-induced *Spic* in lungs (Figure 6D). Based on these findings, we wondered whether some of the known anti-inflammatory effects of blocking IFNγ during inflammation might be mediated by higher *Spic*. Therefore, we compared inflammatory markers in WT and *Spic*$^{-/-}$ mice treated with LPS and anti- IFNγ and found higher levels of inflammatory markers in the absence of *Spic* (Figure 6E).

We next asked whether IFNγ could also suppress *Spic* in macrophages that already express high levels of this transcription factor at the steady state (non-TLR induced). Mice treated with recombinant IFNγ showed a reduced expression of *Spic* and *Fpn* in RPMs (Figure 6F). To confirm that a lower *Spic* expression in this setting reflects the direct action of IFNγ within macrophages, we isolated RPM and exposed them to IFNγ *ex vivo*, which reduced *Spic* (Figure 6G). IFNγ also suppressed heme-induced *Spic* in BMDMs (Figure 6H). Indeed, IFNγ alone further suppressed the very low levels of basal *Spic* and *Fpn* in BMDMs (Figure S5C). Hence, the suppressive effects of IFNγ on *Spic* is independent of the stimuli inducing *Spic* expression.

Spic Induction in Sterile Inflammation

NF-κB is also activated in macrophages in PAMP-independent sterile inflammation. Because sterile inflammation is usually not associated with high IFNs, we wondered whether this might also be a relevant setting of *Spic* induction and function. We first examined whether *Spic* is induced in lung macrophages upon bleomycin exposure, a commonly used model of sterile lung inflammation and fibrosis (Liu et al., 2017). We found significantly elevated *Spic* in lungs of bleomycin-treated mice compared to controls (Figure 7A). *Spic* expression was higher at later time points, suggestive of its role during the resolution stage of the injury (Figure 7A). We also examined *Spic* induction in a different type of sterile inflammation within a different organ, namely, ischemia-reperfusion injury in kidney (Aufhauser et al., 2016). Consistent with our observation in lungs, *Spic* was significantly elevated in the kidneys 30 days post-injury (Figure 7B). These findings show that sterile inflammation can also induce *Spic*.

Tissue macrophages are heterogeneous in origin (monocyte derived versus embryonic) and phenotype. As an example, lungs contain SiglecF^{hi}CD11c^{hi}CD11B^{-} alveolar macrophages (embryonic origin), SiglecF^{-}CD11c^{-}CD11B^{+} MHCII^{hi} interstitial macrophages (monocyte derived), and SiglecF^{-}CD11c^{-}CD11B^{+} MHCII^{low} interstitial macrophages (monocyte derived) (Chakarov et al., 2019). We next examined whether pathogen-associated and sterile inflammation induce *Spic* in specific macrophage subsets within the same tissue by using lung as the model. Intra-peritoneal LPS induced *Spic* predominantly in interstitial macrophages, whereas intra-tracheal bleomycin induced it in both alveolar and interstitial macrophages in the lungs (Figure S6). A confounding factor in this analysis is that exposure to PAMPs and DAMPs may alter the expression of key surface markers on macrophages, which can make it difficult to identify the different macrophage subsets. To circumvent this limitation, we used a genetic model where *Spic* expression does not rely on PAMP or

DAMP activation. As described above, *Bach1* is a negative regulator of *Spic*, and *Bach1*-deficient mice constitutively express high levels of *Spic* in macrophages. Examination of lung macrophages in *Bach1*$^{-/-}$ *Spic*$^{GFP/GFP}$ mice clearly showed *Spic* in both alveolar and interstitial macrophages (Figures S7A and S7B). Hence, all major macrophage subsets in the lungs appear capable of inducing *Spic* with appropriate stimuli.

Next, we examined the pathophysiological implications of *Spic* induction in sterile inflammation. Bleomycin-induced lung injury appeared to engender a stronger fibrotic response in *Spic*$^{-/-}$ mice than in WT mice based on the expression of collagen 1a1 and Tenascin C, two markers of lung fibrosis (Figure 7C). Finally, we asked whether sterile-inflammation-associated human pathological conditions may be associated with macrophage *Spic* expression by analyzing a public dataset of single-cell RNA sequencing of renal immune cells from normal and lupus nephritis patients (Arazi et al., 2019). Consistent with our observations in mice, a subset of monocyte and Mo-MAC in nephritic, but not normal, kidney expressed high levels of *Spic* (Figure S7C).

In summary, we provide evidence of a transcriptional circuitry by which macrophages sense and respond to their inflammatory milieu (Figure 7D). At the core of this mechanism lies counter-regulation of *Spic* by NF-κB and STAT (IFN signaling). NF-κB is activated in myriad settings in various cell types; yet, the induction of *Spic* is highly restricted to patrolling monocytes and macrophages, highlighting a lineage-restricted role of this pathway.

DISCUSSION

Activated macrophages release effector molecules that not only control infection but also cause tissue damage. Therefore, macrophage inflammatory responses are downregulated after elimination of the infectious threat. Indeed, macrophages undergo a switch from a pro- to anti-inflammatory phenotype during the resolution of inflammation. Our findings support a role of the transcription factor *Spic* in facilitating this switch. Although the role of macrophages in systemic iron homeostasis is well known, there is also a growing appreciation of their importance in regulating local iron availability (Winn et al., 2020). Iron is an essential element in many key biological processes, and hence, local iron availability can affect tissue homeostasis. As an example, iron efflux from macrophages was shown to influence tissue repair in the skin (Recalcati et al., 2019). How tissue repair is regulated by local macrophage iron efflux likely depends on the tissue type and/or the nature of the injury, and our study shows that the transcription factor *Spic* may have a key role in this process.

Two recent studies reported the induction of *Spic* in inflammation-induced hemophagocytes, which are monocyte-derived cells that phagocytose red cells and other leukocytes (Akilesh et al., 2019; Wang et al., 2019). These hemophagocytes are thought to drive inflammation and cytopenia. Our findings are congruent with these recent reports and extend the field by uncovering the function and regulation of *Spic* in these inflammatory settings. Furthermore, *Spic* induction in sterile inflammation and its negative regulation by IFNγ indicate a general role of this transcriptional circuit within activated macrophages.

An intriguing observation in our study is the highly restricted nature of *Spic* expression. The capacity to induce this transcription factor was restricted to macrophages but not DCs, whereas its expression in monocytes was restricted to the patrolling subset. Although the molecular basis of this specific expression pattern awaits further studies, it underscores the functional distinction between monocyte subsets.

The requirement of NF-κB for macrophage *Spic* expression is consistent with a previous study that describes a role of NF-κB in *Spic* expression during B cell development (Bednarski et al., 2016). Macrophages activate NF-κB in response to many other stimuli besides TLR activation. Therefore, it was somewhat surprising that the role of *Spic* in activated macrophages has not been widely reported. One explanation is the existence of counter-regulatory mechanisms, one of which (IFN dependent) we describe here. It is likely that such inhibitory pathways allow *Spic* expression only in situations where the threat of infection is very low. This type of counter-regulatory mechanism for *Spic* expression allows fine-tuning of macrophage inflammatory responses.

STAR★METHODS

Detailed methods are provided in the online version of this paper and include the following:

- KEY RESOURCES TABLE
- RESOURCE AVAILABILITY
  - Lead Contact
  - Materials Availability
  - Data and Code Availability
- EXPERIMENTAL MODELS AND SUBJECT DETAILS
  - Mice
  - Inflammation models
  - Cell culture
- METHOD DETAILS
  - Tissue harvest and flow cytometry
  - Gene expression profiling by microarray
  - Quantitative real-time PCR (qRT-PCR)
  - Iron Quantitation
  - Measurement of cytokines by ELISA
- QUANTIFICATION AND STATISTICAL ANALYSIS

SUPPLEMENTAL INFORMATION

Supplemental Information can be found online at https://doi.org/10.1016/j.celrep.2020.107825.

ACKNOWLEDGMENTS

This work was supported by National Institutes of Health grant NIAID-KO8AI106953 (M.H.), a Burroughs Wellcome Fund (BWF) Career Awards for Medical Scientists (CAMS) (M.H.), and an American Society of Hematology (ASH) scholar award (M.H.). We thank Dr. Hsiou-chi Liou for providing the *c-Rel* knockout mice.

AUTHOR CONTRIBUTIONS

Conceptualization and Methodology, M.H., Z.A., S.D., M.L., and M.T.D.; Analysis, Z.A., M.H., S.D., M.T.D., T.K.J.T., I.W.F., and M.A.S.; Investigation, Z.A.,

M.H., M.A.S., M.T.D., Y.H.C., X.L., C.J.W., P.T.H., A.B., M.B., M.H.L., E.M.-V., and I.W.F.; Resources, I.W.F., M.H., M.T.D., P.Y., M.A.S., and M.L.; Data Curation, M.H., Z.A., S.D., M.L., M.T.D., P.Y., I.W.F., M.A.S., and J.T.; Writing, Z.A. and M.H.; Editing, S.D., I.W.F., M.A.S., J.T., X.L., C.J.W., and Y.H.C.; Supervision, M.H.; Project Administration, M.H.

DECLARATION OF INTERESTS

The authors declare no competing interests.

Received: August 26, 2019
Revised: March 27, 2020
Accepted: June 5, 2020
Published: June 30, 2020

REFERENCES

Abreu, R., Quinn, F., and Giri, P.K. (2018). Role of the hepcidin-ferroportin axis in pathogen-mediated intracellular iron sequestration in human phagocytic cells. Blood Adv. *2*, 1089–1100.

Akilesh, H.M., Buechler, M.B., Duggan, J.M., Hahn, W.O., Matta, B., Sun, X., Gessay, G., Whalen, E., Mason, M., Presnell, S.R., et al. (2019). Chronic TLR7 and TLR9 signaling drives anemia via differentiation of specialized hemophagocytes. Science *363*, eaao5213.

Alam, M.Z., Devalaraja, S., and Haldar, M. (2017). The Heme Connection: Linking Erythrocytes and Macrophage Biology. Front. Immunol. *8*, 33.

Arazi, A., Rao, D.A., Berthier, C.C., Davidson, A., Liu, Y., Hoover, P.J., Chicoine, A., Eisenhaure, T.M., Jonsson, A.H., Li, S., et al.; Accelerating Medicines Partnership in SLE network (2019). The immune cell landscape in kidneys of patients with lupus nephritis. Nat. Immunol. *20*, 902–914.

Aufhauser, D.D., Jr., Wang, Z., Murken, D.R., Bhatti, T.R., Wang, Y., Ge, G., Redfield, R.R., III, Abt, P.L., Wang, L., Svoronos, N., et al. (2016). Improved renal ischemia tolerance in females influences kidney transplantation outcomes. J. Clin. Invest. *126*, 1968–1977.

Bain, C.C., Scott, C.L., Uronen-Hansson, H., Gudjonsson, S., Jansson, O., Grip, O., Guilliams, M., Malissen, B., Agace, W.W., and Mowat, A.M. (2013). Resident and pro-inflammatory macrophages in the colon represent alternative context-dependent fates of the same Ly6Chi monocyte precursors. Mucosal Immunol. *6*, 498–510.

Bednarski, J.J., Pandey, R., Schulte, E., White, L.S., Chen, B.-R., Sandoval, G.J., Kohyama, M., Haldar, M., Nickless, A., Trott, A., et al. (2016). RAG-mediated DNA double-strand breaks activate a cell type-specific checkpoint to inhibit pre-B cell receptor signals. J. Exp. Med. *213*, 209–223.

Briseño, C.G., Haldar, M., Kretzer, N.M., Wu, X., Theisen, D.J., Kc, W., Durai, V., Grajales-Reyes, G.E., Iwata, A., Bagadia, P., et al. (2016). Distinct Transcriptional Programs Control Cross-Priming in Classical and Monocyte-Derived Dendritic Cells. Cell Rep. *15*, 2462–2474.

Chakarov, S., Lim, H.Y., Tan, L., Lim, S.Y., See, P., Lum, J., Zhang, X.-M., Foo, S., Nakamizo, S., Duan, K., et al. (2019). Two distinct interstitial macrophage populations coexist across tissues in specific subtissular niches. Science *363*, eaau0964.

Drakesmith, H., and Prentice, A.M. (2012). Hepcidin and the iron-infection axis. Science *338*, 768–772.

Drakesmith, H., Nemeth, E., and Ganz, T. (2015). Ironing out Ferroportin. Cell Metab. *22*, 777–787.

Ganz, T., and Nemeth, E. (2015). Iron homeostasis in host defence and inflammation. Nat. Rev. Immunol. *15*, 500–510.

Geissmann, F., Jung, S., and Littman, D.R. (2003). Blood monocytes consist of two principal subsets with distinct migratory properties. Immunity *19*, 71–82.

Gordon, S., Plüddemann, A., and Martinez Estrada, F. (2014). Macrophage heterogeneity in tissues: phenotypic diversity and functions. Immunol. Rev. *262*, 36–55.

Guida, C., Altamura, S., Klein, F.A., Galy, B., Boutros, M., Ulmer, A.J., Hentze, M.W., and Muckenthaler, M.U. (2015). A novel inflammatory pathway mediating rapid hepcidin-independent hypoferremia. Blood *125*, 2265–2275.

Haldar, M., and Murphy, K.M. (2014). Origin, development, and homeostasis of tissue-resident macrophages. Immunol. Rev. *262*, 25–35.

Haldar, M., Kohyama, M., So, A.Y.-L., Kc, W., Wu, X., Briseño, C.G., Satpathy, A.T., Kretzer, N.M., Arase, H., Rajasekaran, N.S., et al. (2014). Heme-mediated SPI-C induction promotes monocyte differentiation into iron-recycling macrophages. Cell *156*, 1223–1234.

Harusato, A., Naito, Y., Takagi, T., Uchiyama, K., Mizushima, K., Hirai, Y., Higashimura, Y., Katada, K., Handa, O., Ishikawa, T., et al. (2013). BTB and CNC homolog 1 (Bach1) deficiency ameliorates TNBS colitis in mice: role of M2 macrophages and heme oxygenase-1. Inflamm. Bowel Dis. *19*, 740–753.

Helft, J., Böttcher, J., Chakravarty, P., Zelenay, S., Huotari, J., Schraml, B.U., Goubau, D., and Reis e Sousa, C. (2015). GM-CSF Mouse Bone Marrow Cultures Comprise a Heterogeneous Population of CD11c(+)MHCII(+) Macrophages and Dendritic Cells. Immunity *42*, 1197–1211.

Hu, X., and Ivashkiv, L.B. (2009). Cross-regulation of signaling pathways by interferon-gamma: implications for immune responses and autoimmune diseases. Immunity *31*, 539–550.

Igarashi, K., and Watanabe-Matsui, M. (2014). Wearing red for signaling: the heme-bach axis in heme metabolism, oxidative stress response and iron immunology. Tohoku J. Exp. Med. *232*, 229–253.

Kaneda, M.M., Messer, K.S., Ralainirina, N., Li, H., Leem, C.J., Gorjestani, S., Woo, G., Nguyen, A.V., Figueiredo, C.C., Foubert, P., et al. (2016). PI3Kγ is a molecular switch that controls immune suppression. Nature *539*, 437–442.

Kayama, H., Kohyama, M., Okuzaki, D., Motooka, D., Barman, S., Okumura, R., Muneta, M., Hoshino, K., Sasaki, I., Ise, W., et al. (2018). Heme ameliorates dextran sodium sulfate-induced colitis through providing intestinal macrophages with noninflammatory profiles. Proc. Natl. Acad. Sci. USA *115*, 8418–8423.

Kohyama, M., Ise, W., Edelson, B.T., Wilker, P.R., Hildner, K., Mejia, C., Frazier, W.A., Murphy, T.L., and Murphy, K.M. (2009). Role for Spi-C in the development of red pulp macrophages and splenic iron homeostasis. Nature *457*, 318–321.

Krutzik, S.R., Tan, B., Li, H., Ochoa, M.T., Liu, P.T., Sharfstein, S.E., Graeber, T.G., Sieling, P.A., Liu, Y.-J., Rea, T.H., et al. (2005). TLR activation triggers the rapid differentiation of monocytes into macrophages and dendritic cells. Nat. Med. *11*, 653–660.

Liu, T., De Los Santos, F.G., and Phan, S.H. (2017). The Bleomycin Model of Pulmonary Fibrosis. Methods Mol. Biol. *1627*, 27–42.

Ma, Q. (2013). Role of nrf2 in oxidative stress and toxicity. Annu. Rev. Pharmacol. Toxicol. *53*, 401–426.

Medzhitov, R., and Horng, T. (2009). Transcriptional control of the inflammatory response. Nat. Rev. Immunol. *9*, 692–703.

Mukhopadhyay, S., Plüddemann, A., and Gordon, S. (2009). Macrophage pattern recognition receptors in immunity, homeostasis and self tolerance. Adv. Exp. Med. Biol. *653*, 1–14.

Murray, P.J. (2017). Macrophage Polarization. Annu. Rev. Physiol. *79*, 541–566.

Murray, P.J., and Wynn, T.A. (2011). Protective and pathogenic functions of macrophage subsets. Nat. Rev. Immunol. *11*, 723–737.

Oishi, Y., and Manabe, I. (2018). Macrophages in inflammation, repair and regeneration. Int. Immunol. *30*, 511–528.

Recalcati, S., Gammella, E., Buratti, P., Doni, A., Anselmo, A., Locati, M., and Cairo, G. (2019). Macrophage ferroportin is essential for stromal cell proliferation in wound healing. Haematologica *104*, 47–58.

Ruffell, D., Mourkioti, F., Gambardella, A., Kirstetter, P., Lopez, R.G., Rosenthal, N., and Nerlov, C. (2009). A CREB-C/EBPbeta cascade induces M2

macrophage-specific gene expression and promotes muscle injury repair. Proc. Natl. Acad. Sci. USA *106*, 17475–17480.

Satpathy, A.T., Kc, W., Albring, J.C., Edelson, B.T., Kretzer, N.M., Bhattacharya, D., Murphy, T.L., and Murphy, K.M. (2012). Zbtb46 expression distinguishes classical dendritic cells and their committed progenitors from other immune lineages. J. Exp. Med. *209*, 1135–1152.

Shaw, T.N., Houston, S.A., Wemyss, K., Bridgeman, H.M., Barbera, T.A., Zangerle-Murray, T., Strangward, P., Ridley, A.J.L., Wang, P., Tamoutounour, S., et al. (2018). Tissue-resident macrophages in the intestine are long lived and defined by Tim-4 and CD4 expression. J. Exp. Med. *215*, 1507–1518.

Soares, M.P., and Hamza, I. (2016). Macrophages and Iron Metabolism. Immunity *44*, 492–504.

Thäle, C., and Kiderlen, A.F. (2005). Sources of interferon-gamma (IFN-gamma) in early immune response to Listeria monocytogenes. Immunobiology *210*, 673–683.

Wang, A., Pope, S.D., Weinstein, J.S., Yu, S., Zhang, C., Booth, C.J., and Medzhitov, R. (2019). Specific sequences of infectious challenge lead to secondary hemophagocytic lymphohistiocytosis-like disease in mice. Proc. Natl. Acad. Sci. USA *116*, 2200–2209.

Winn, N.C., Volk, K.M., and Hasty, A.H. (2020). Regulation of tissue iron homeostasis: the macrophage "ferrostat". JCI Insight *5*, e132964.

Wynn, T.A., and Vannella, K.M. (2016). Macrophages in Tissue Repair, Regeneration, and Fibrosis. Immunity *44*, 450–462.

Zhang, X., and Mosser, D.M. (2008). Macrophage activation by endogenous danger signals. J. Pathol. *214*, 161–178.

STAR★METHODS

KEY RESOURCES TABLE

REAGENT or RESOURCE	SOURCE	IDENTIFIER
Antibodies		
Anti-mouse-PECY7-CD11b antibody	Invitrogen	Cat# 25-0112-82
Anti-mouse-APC/CY7-CD115 antibody	Biolegend	Cat# 135531
Anti-mouse-AF647-CD64 antibody	BD Bioscience	Cat# 558539
Anti-mouse-PE-CD103 antibody	Biolegend	Cat# 121406
Anti-mouse-BV510-Ly6G antibody	Biolegend	Cat# 127633
Anti-mouse-APC-CD115 antibody	eBioscience	Cat# 17-1152-82
Anti-mouse-APC/CY7-CD11C antibody	Biolegend	Cat# 117324
Anti-mouse-BV510-CD45 antibody	Biolegend	Cat# 103138
Anti-mouse-APC/CY7-Ly6G antibody	Biolegend	Cat# 127624
Anti-mouse-BV510-MHC II antibody	Biolegend	Cat# 107635
Anti-mouse-APC-CD11b antibody	Biolegend	Cat# 101212
Anti-mouse-AF488-F4/80 antibody	Bio Rad	Cat# MCA497A488
Anti-mouse-Percpcy5.5-Ly6G antibody	Biolegend	Cat# 127615
Anti-mouse-APC-F4/80 antibody	Invitrogen	Cat# MF48005
Anti-mouse-PECY7-MerTK antibody	eBioscience	Cat# 25-575-82
Anti-mouse-BV605-CD45 antibody	Biolegend	Cat# 103139
Anti-mouse-PECY7-CD301 antibody	Biolegend	Cat# 145706
Anti-mouse-AF488-CD45 antibody	Biolegend	Cat# 103122
Purified from rabbit-PE- FPN- antibody	Novus	Cat# F-1-062918-PE
Anti-mouse-PE-TremL4-antibody	Biolegend	Cat# 143304
Anti-mouse-BV421-CD206 antibody	Biolegend	Cat# 141717
Anti-mouse-BV605-Ly6C antibody	Biolegend	Cat# 128036
Anti-mouse-Percpcy5.5-CD45 antibody	Biolegend	Cat# 103132
Anti-mouse-BV605-CD11b antibody	Biolegend	Cat# 101257
Anti-mouse-APC/CY7-Ly6C antibody	Biolegend	Cat# 128026
Anti-mouse-BV421-MHC II antibody	Biolegend	Cat# 107631
Anti-mouse-BV421-SiglecF antibody	BD Biosciences	Cat# 562681
Anti-mouse-APC-SiglecF antibody	Biolegend	Cat# 155508
Anti-mouse-PECY7-Tim-4 antibody	Biolegend	Cat# 130009
Anti-mouse-BV421-CD4 antibody	Biolegend	Cat# 100437
Ultra-LEAF Purified anti-mouse IFN-γ Antibody	BioLegend	Cat# 505847
Chemicals, Peptides, and Recombinant Proteins		
Lipopolysaccharide	Sigma-Aldrich	Cat# L4391-10X1MG
Bleomycin	Fresenius Kabi	DIN: 02265982
BOT-64, IKK-2 inhibitor	Fisher Scientific	Cat# AAJ64555LB0
T-5224 (AP-1 Inhibitor)	Cayman Chemical	Cat# 22904
Phosphate Buffered Saline (PBS)	Life Technologies	Cat# 13151014
UltraPure 0.5M EDTA	Invitrogen	Cat# 15575-038
Penicillin-Streptomycin (10,000 U/mL)	Thermo Fisher	Cat# 15140122
L-Glutamine	Thermo Fisher	Cat# 25030081
GIBCO-Non Essential Amino Acid	Fisher Scientific	Cat# 11140050
Sodium Pyruvate (100 mM)	Thermo Fisher	Cat# 11360070
2-mercaptoethanol	Fisher Scientific	Cat# 21985023
Fludarabine 10mM	Selleck chemicals	Cat# S1491

(*Continued on next page*)

Continued

REAGENT or RESOURCE	SOURCE	IDENTIFIER
7AAD Viability staining	Biolegend	Cat# 420404
Murine GM-CSF	PeproTech	Cat# 315-03-20ug
Murine M-CSF	PeproTech	Cat# 315-02-50ug
Murine INF-gamma	PeproTech	Cat# 315-05-100ug
TaqMan Universal Master Mix	Thermo Fisher	Cat# 4304437
Critical Commercial Assays		
TNF alpha ELISA Kit, Mouse	Thermo Fisher	Cat# BMS607-3
Monocyte Isolation Kit (BM), mouse	Miltenyi Biotec	Cat# 130-100-629
High Capacity RNA to cDNA kit	Thermo Fisher	Cat# 4387406
Genelute Mammalian Total RNA isolation kit	Sigma-Aldrich	Cat# RTN70-1KT
Iron assay kit	Abcam	Cat# ab83366
Deposited Data		
Microarray data	This study	GSE150520
Experimental Models: Organisms/Strains		
Mouse: Spic-Knockout	Kohyama et al., 2009	PMID: 19037245
Mouse: Spic-GFP	Haldar et al., 2014	PMID: 24630724
Mouse: Bach1-Knockout	EUCOMM	MGI:5009633
Mouse: cREL-Knockout	Dr. Hsiou-Chi Liou	PMID: 12235116
Oligonucleotides		
SpiC (TaqMan primers and probe)	Thermo Fisher	Mm00488428_m1
Arg1 (TaqMan primers and probe)	Thermo Fisher	Mm00475988_m1
IL10 (TaqMan primers and probe)	Thermo Fisher	Mm01288386_m1
TNFalpha (TaqMan primers and probe)	Thermo Fisher	Mm00443258_m1
NOS2 (TaqMan primers and probe)	Thermo Fisher	Mm00440502_m1
IL-1beta (TaqMan primers and probe)	Thermo Fisher	Mm00434228_m1
IL-6 (TaqMan primers and probe)	Thermo Fisher	Mm00446190_m1
Bach1 (TaqMan primers and probe)	Thermo Fisher	Mm01344527_m1
Ferroportin (Slc40a1) (TaqMan primers and probe)	Thermo Fisher	Mm01254822_m1
Fth1 (TaqMan primers and probe)	Thermo Fisher	Mm00850707_g1
Ftl1 (TaqMan primers and probe)	Thermo Fisher	Mm03030144_g1
Nrf2 (TaqMan primers and probe)	Thermo Fisher	Mm04231240_s1
Hamp (TaqMan primers and probe)	Thermo Fisher	Mm00477784_m1
Hmox1 (TaqMan primers and probe)	Thermo Fisher	Mm00516005_m1
Col1a1 (TaqMan primers and probe)	Thermo Fisher	Mm00801666_g1
Tnc (TaqMan primers and probe)	Thermo Fisher	Mm00495662_m1
HPRT (TaqMan primers and probe)	Thermo Fisher	Mm03024075_m1
Euk 18S rRNA (TaqMan primers and probe)	Life Technologies	Cat# 4333760F
Software and Algorithms		
GraphPad Prism 8	Graph Pad Software	N/A
FlowJo LLC V10.1	FlowJo LLC	N/A
Gene Set Enrichment Analysis (GSEA)	Broad Institute	N/A
Arraystar 4	DNASTAR	N/A
Other		
Iscove's Modified Dulbecco's Medium (IMDM)	Thermo Fisher	Cat# 12440053
Dulbecco's Modified Eagle Medium (DMEM)	Thermo Fisher	Cat# 10567014
Bovine Serum Albumin (BSA)	VWR	Cat# 97061-420
Fetal Bovine Serum (FBS)	GeminiBio	Cat#100-500
Collagenase B	Roche	Cat# 11088831001
DNase I	Sigma-Aldrich	Cat# D4527-40KU

RESOURCE AVAILABILITY

Lead Contact

Requests for additional information about the manuscript or for resources and reagents should be directed to and will be fulfilled by the Lead Contact, Malay Haldar (mhaldar@pennmedicine.upenn.edu).

Materials Availability

This study did not generate new unique reagents

Data and Code Availability

The accession number for the micarroarray data described in this manuscript isGEO: GSE150520.

EXPERIMENTAL MODELS AND SUBJECT DETAILS

Mice

Spic$^{-/-}$ and *Spic*$^{GFP/GFP}$ mice were described before (Haldar et al., 2014; Kohyama et al., 2009). *Bach1*$^{-/-}$ were generated by the European Conditional Mouse Mutagenesis Program (EUCOMM). *c-rel*$^{-/-}$ mice were kindly provided by Dr. Youhai H. Chen from the University of Pennsylvania. *Spic*$^{-/-}$ mice are in 129/SvEv and *Spic*$^{GFP/GFP}$ mice in C57/6J background. Both male and female mice between 2-12 months of age were used in the experiments.

Mice were genotyped using published primer sets and PCR protocol. Germ-free C57BL/6J mice were obtained from the PennCHOP Microbiome Program Gnotobiotic Core facility. The university of Pennsylvania Institutional Animal Care and Use Committee approved all mouse experiments.

Inflammation models

For pathogen-associated inflammation, *Escherichia coli*-derived lipopolysaccharides were injected intraperitoneally (100-150 μg of LPS in sterile1X PBS, 200 μL total volume). For IFNγ blockade, 200 μg of anti-mouse IFNγ antibody in 200 μL of total volume was injected (intraperitoneal).

For sterile pulmonary inflammation, bleomycin (at 3U/kg; Fresenius Kabi) or water was instilled intratracheally in wild-type C57BL/6 mice. The mice were euthanized at various time points after injury and the lungs harvested and processed for downstream experiments.

For inducing kidney ischemia reperfusion injury (IRI), mice were anesthetized with pentobarbital sodium (65 mg/kg IP) and placed in a temperature-controlled operative apparatus. Core body temperature was continuously measured and maintained at 36.0 ± 0.5°C. Under an operating microscope, the left renal pedicle exposed and clamped for 28 min with a microvascular clip (Roboz Surgical Instrument, Gaithersburg, MD). After the clamp was released, the right kidney was exposed and removed. After closure, animals were subcutaneously injected with 100 mL/kg of warm saline after the operation to ensure hydration. Animals were kept in an incubator (37°C) until awake. Mice were given access to water ad-lib post-procedure. All animal protocols adhered to the NIH Guide for the Care and Use of Laboratory Animals and were performed in an AAALAC accredited facility.

Cell culture

Bone marrow derived macrophage (BMDM): Total bone marrow cells were flushed out of femur, red cells removed using RBC lysis buffer, and remaining cells cultured in Iscove's Modified Dulbecco's Medium (IMDM) containing 10% FCS and supplemented with 20 ng/ml M-CSF. Macrophages were generated after 7-9 days in this culture.

Monocyte-derived macrophages (Mo-MACs): Monocytes were isolated from bone marrow cells using the 'monocyte-isolation kit (BM)' from Miltenyi Biotech and following manufacturer's protocol. Monocytes were then cultured in IMDM + 10%FCS supplemented with M-CSF to generate Mo-MACs after 3-5 days in culture.

In vitro treatments: Cell culture media from BMDMs or Mo-MACs were removed and replaced with fresh media (without M-CSF) containing TLR ligands and/or drugs at indicated doses. TLR ligands: LPS (100 to 1000 ng/ml) and CpG (30 μg/ml), IKK-2 inhibitor (10 μM), cycloheximide (10 μg/ml), AP1 inhibitor (10 μM), STAT1 blocker (50 μM), and PI3K-gamma inhibitor (100 nM).

METHOD DETAILS

Tissue harvest and flow cytometry

Organs were harvested from euthanized mice, washed with sterile PBS, and cut into small pieces (1-3 mm). Tissue pieces were then digested with an enzyme cocktail (5 ml) comprised of DMEM (with 10% FBS) containing collagenase at 0.25 mg/ml (Roche) and DNase I at 30 U/ml (Sigma-Aldrich). Tissue digestion occurred at 37°C for 45 min with constant stirring. After the digestion, the materials were filtered through 70-μm nylon filter (Celltreat Scientific Product), RBC lysed, and single-cell suspensions.

For flow cytometry, cells were counted and incubated with fluorescently tagged antibodies in MACS buffer (1X PBS, 0.5 mM EDTA, and 0.5% BSA).

Gene expression profiling by microarray

Microarray was performed at the UPenn Molecular Profiling Facility, including quality control tests of the total RNA samples by agilent bioanalyzer and nanodrop spectrophotometry. All protocols were conducted as described in the Affymetrix WT Pico Reagent Kit Manual and the Affymetrix GeneChip Expression Analysis Technical Manual. Gene expression data were normalized and values modeled using ArrayStar4 (DNASTAR). Microarray reported here is deposited in Gene Expression Omnibus (GEO: GSE150520).

Quantitative real-time PCR (qRT-PCR)

Total RNA was isolated from tissues and cells using the GenElute Mammalian Total RNA Miniprep Kit (Sigma-Aldrich) or RNeasy Mini Kit (QIAGEN). Reverse transcription of mRNA was performed using the High-Capacity RNA-to-cDNA Kit (Life Technologies). qRT-PCR was performed using a ViiA7 Real-Time PCR system. All probes were obtained from TaqMan.

Iron Quantitation

BMDMs were generated in 75 mm flask in I-10F media (10 mL), containing 20 ng/mL of M-CSF. After 7 days of culture, the media was replaced with 10 mL of fresh I-10F media containing 100 mM of FeSO4 (Sigma Aldrich F8633). Cells were then treated with LPS (1000 ng/mL) or control (PBS). 24 h later the media was removed and the adherent BMDMs were washed 3X with sterile ice-cold PBS. BMDMs were then detached with trypsin (GIBCO, 0.25%) and pelleted by centrifugation at 450xg for 5 min. Supernatant was removed and the cells re-suspended in 1 mL of IL-10F media and cell numbers counted to ensure similar numbers of cells in each assay condition. The cell suspension were spun down again (450xg for 5 min) and re-suspended in 400 uL of Iron assay buffer. Cells were next sonicated (1 min/sample) and spun down at 1300xg for 5 min. The supernatant was collected and stored in −80 freezer. Iron assay was performed on the stored supernatant using the Iron Assay Kit (Abcam, catalog no: 83366) and following the manufacturer’s protocol.

Measurement of cytokines by ELISA

Cell culture supernatant were collected and stored in −80°C until cytokines concentrations were quantified by ELISA. By following the protocol as provided by the manufacturer, the concentrations of TNFα and IL1β were measured using the mouse TNF alpha and IL1 beta ELISA Kit.

QUANTIFICATION AND STATISTICAL ANALYSIS

To calculate the significance for two individual groups, unpaired t test were performed. To compare the mean of three or more groups, one-way ANOVA with Tukey’s multiple comparison tests were used. p values of < 0.05 (*), < 0.01 (**), < 0.001 (***) and < 0.0001 (****) were considered statistically significant. Statistically non-significant is indicated as ns. Data were analyzed using the GraphPad Prism Software (Prism 5).

Cell Reports, Volume *31*

Supplemental Information

Counter Regulation of *Spic* by NF-κB and STAT Signaling Controls Inflammation and Iron Metabolism in Macrophages

Zahidul Alam, Samir Devalaraja, Minghong Li, Tsun Ki Jerrick To, Ian W. Folkert, Erick Mitchell-Velasquez, Mai T. Dang, Patricia Young, Christopher J. Wilbur, Michael A. Silverman, Xinyuan Li, Youhai H. Chen, Paul T. Hernandez, Aritra Bhattacharyya, Mallar Bhattacharya, Matthew H. Levine, and Malay Haldar

Figure S1

A

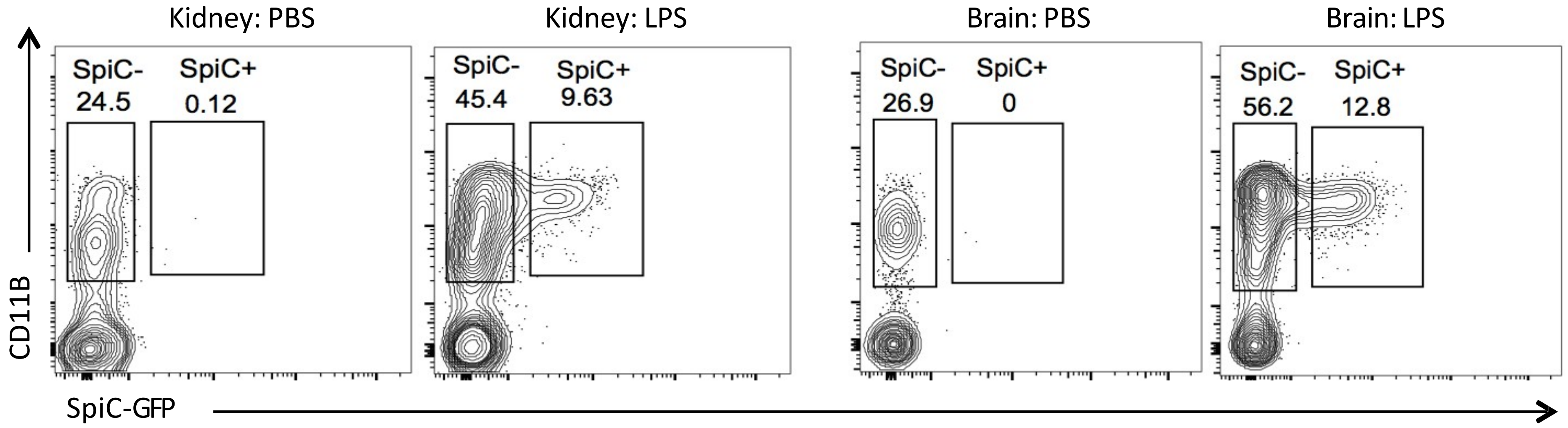
Kidney: PBS
Kidney: LPS
Brain: PBS
Brain: LPS
SpiC-
24.5
SpiC+
0.12
SpiC-
45.4
SpiC+
9.63
SpiC-
26.9
SpiC+
0
SpiC-
56.2
SpiC+
12.8
CD11B
SpiC-GFP

B

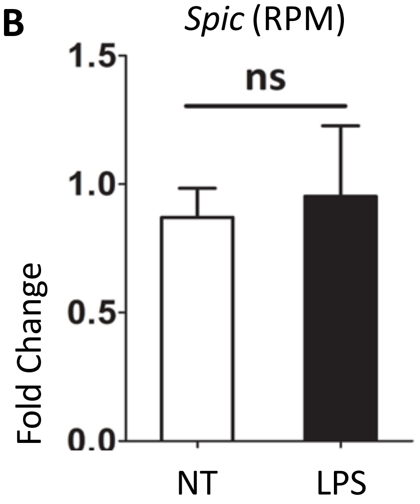
Spic (RPM)
ns
1.5
1.0
0.5
0.0
Fold Change
NT
LPS

Figure S1. **Related to Figure 1 and 2**

(A) *Spic*$^{GFP/GFP}$ mice treated (i.p) with LPS (75 µg in 200 µl PBS) or control (200 µl PBS) and indicated organs harvested 48 hr. after treatment. Shown are the FCS plots with indicated markers on cells pre-gated for CD45+ singlets. **(B)** *Spic*$^{GFP/GFP}$ mice were treated (i.p) with LPS (7.5 µg/gm body weight). 16 hr. after the treatment, RPMs were isolated purified from spleen *via* FACS, RNA extracted, and the expression (normalized to *Hprt*) of *Spic* measured by qRT-PCR. A trend towards higher *Spic* is observed ($P \geq 0.05$). Statistics: unpaired *t*-test. ns: not significant.

FCS: numbers represent percentage of cells within indicated gate. A-D represents ≥3 experiments with ≥3 mice per group. **qRT-PCR**: data representative of ≥3 independent experiments and graphs show single experiment with n≥2 per group. Results expressed as mean ± SEM. NS; not significant.

Figure S2

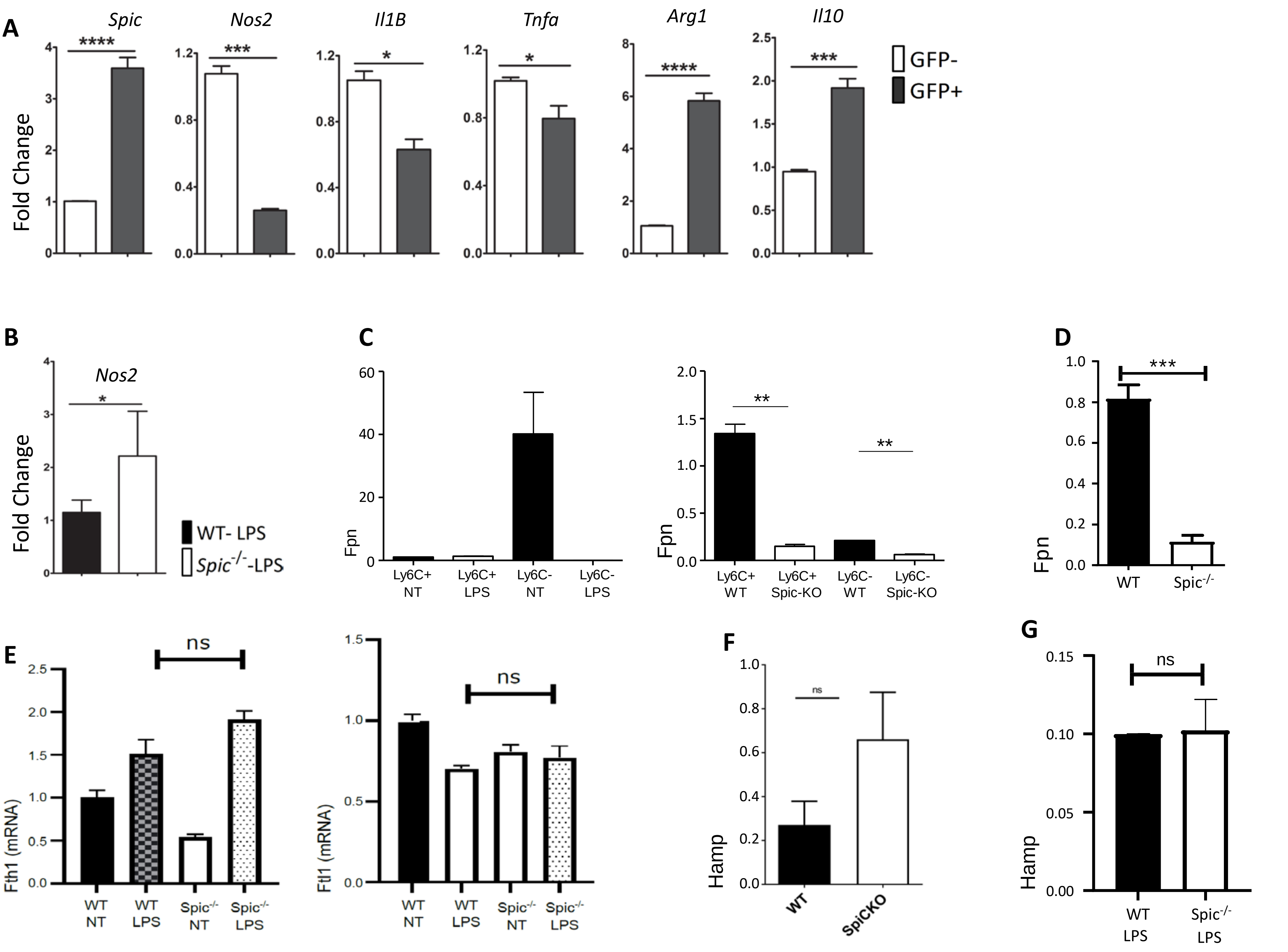

A
Spic
Nos2
Il1B
Tnfa
Arg1
Il10
Fold Change
GFP-
GFP+
****
***
*
*
****
***
B
Nos2
Fold Change
*
WT- LPS
Spic-/--LPS
C
Fpn
Ly6C+ NT
Ly6C+ LPS
Ly6C- NT
Ly6C- LPS
Ly6C+ WT
Ly6C+ Spic-KO
Ly6C- WT
Ly6C- Spic-KO
**
**
D
***
Fpn
WT
Spic-/-
E
ns
Fth1 (mRNA)
Ftl1 (mRNA)
WT NT
WT LPS
Spic-/- NT
Spic-/- LPS
F
ns
Hamp
WT
SpiCKO
G
ns
Hamp
WT LPS
Spic-/- LPS

Figure S2. Related to Figure 3 and 4

(A) *Spic*$^{GFP/GFP}$ BMDMs were treated with LPS (1 µg/ml). 48 hr. later, F4/80^{hi}GFP+ and F4/80^{hi}GFP- cells were purified by FACS, re-plated in media and re-exposed to LPS (1 µg/ml). 2 hr. after LPS re-exposure, RNA was extracted and the expression (normalized to *Hprt)* of indicated genes (Y-axis, relative to GFP- cells) measured by qRT-PCR. **(B)** WT or *Spic*$^{-/-}$ mice were treated (i.p) with LPS (150µg per mouse) twice 48 hr. apart. Lungs were harvested 48 hr. after final LPS treatment and the expression of *Nos2* (normalized to *18S rRNA*) measured (relative to expression in non-treated WT lungs) by qRT-PCR. **(C)** Ly6C^{+}TremL4^{-} classical and Ly6C^{-}TremL4^{+} patrolling monocytes were purified (via FACS) from the peripheral blood of three WT and three *Spic*$^{-/-}$ mice at steady state as well as 72 hr. after treatment (i.p) with LPS (150 µg/mouse). Specified monocyte subsets from each genotype were pooled. Shown is the expression (fold change relative to untreated Ly6C^{+} monocytes) of *Fpn* (relative to *Hprt*). The Left panel shows *Fpn* expression with and without LPS treatment in WT mice; showing that LPS-induced reduction in *Fpn* is largely restricted to Ly6C^{-} monocytes. The right panel compares *Fpn* expression upon LPS treatment in WT and *Spic*$^{-/-}$ monocytes, showing that loss of *Spic* reduces *Fpn* in both monocyte subsets. **(D)** Mice of indicated genotype were treated (i.p) with LPS (100 µg/mouse). Peritoneal fluid (all cells, unfractionated) was collected 24 hr later, RNA extracted, and the expression (normalized to *18S* rRNA) of *Fpn* (Y-axis) measured by qRT-PCR. A combination of three independent experiments is shown (3 mice per group). **(E)** WT and *Spic*$^{-/-}$ Mo-MACs were treated with LPS (100 ng/mL). 24 hr. later, RNA was extracted and the expression (normalized to *Hprt*) of Ferritin heavy chain (*Fth1*) and Ferritin light chain (*Ftl1*) measured by qRT-PCR. **(F)** *Spic*$^{-/-}$ (n=3) and WT (n=5) mice were treated with five doses of intraperitoneal LPS (150µg/mouse per dose) 48 hrs. apart. Liver was harvested 24 hr. after the final LPS treatment, RNA extracted and the expression (relative to *Hprt*) of hepcidin (*Hamp*) measured by qRT-PCR. **(G)** WT and *Spic*$^{-/-}$ Mo-MACs were treated with LPS (100 ng/mL). 24 hr. later, RNA was extracted and the expression (normalized to *Hprt*) of *Hamp* measured by qRT-PCR.

qRT-PCR: data representative of $\geq$3 independent experiments and graphs show single experiment with n$\geq$2 per group. Results expressed as mean ± SEM. $P \leq 0.05$ (*), $P \leq 0.01$ (**), $P \leq 0.001$ (***), and $P \leq 0.0001$ (****), ns; not significant.

Figure S3

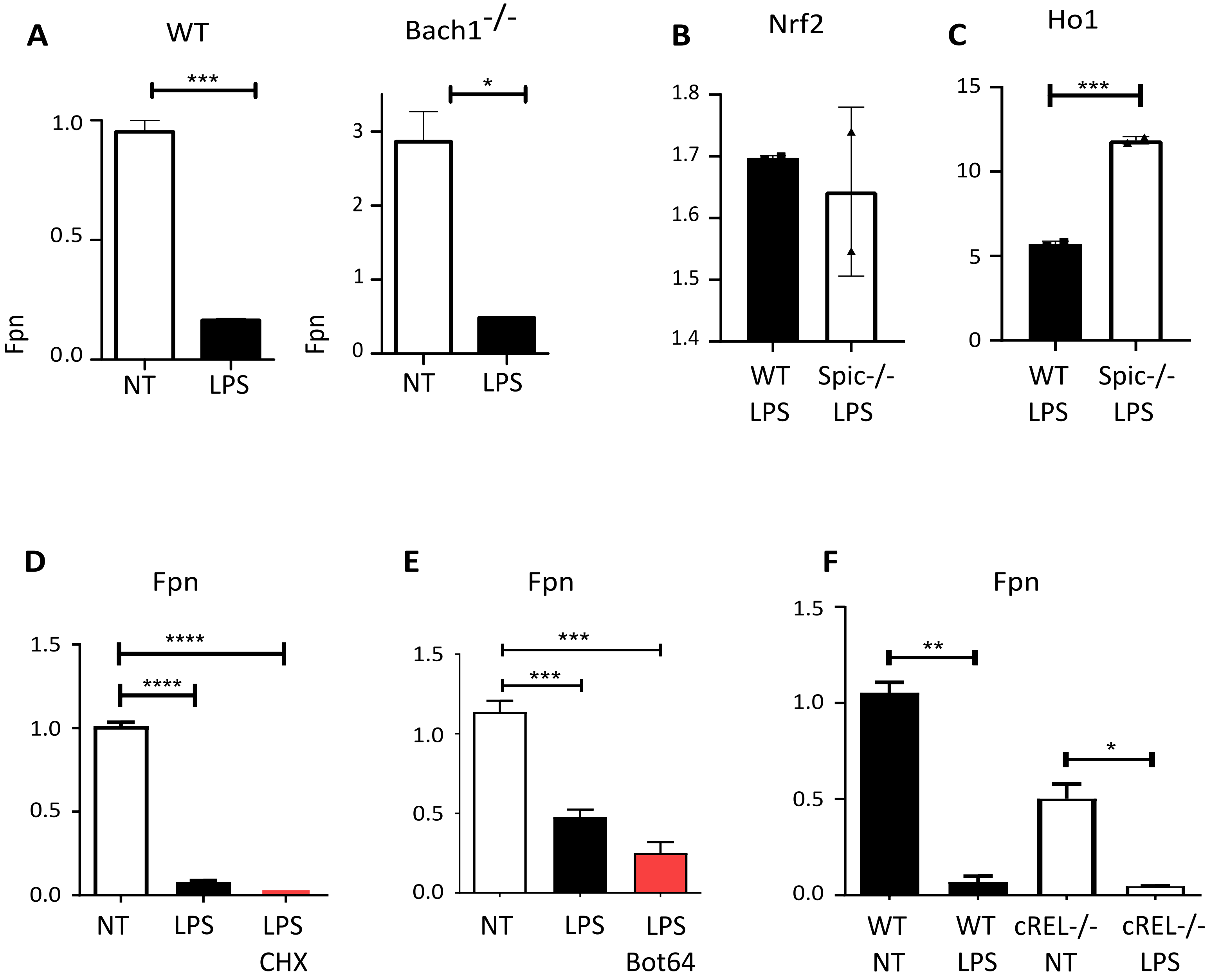
A
WT
Bach1-/-
Fpn
***
*
1.0
0.5
0.0
3
2
1
0
NT
LPS
B
Nrf2
1.8
1.7
1.6
1.5
1.4
WT LPS
Spic-/- LPS
C
Ho1
***
15
10
5
0
D
Fpn
****
****
1.5
1.0
0.5
0.0
NT
LPS
LPS CHX
E
Fpn
***
***
LPS Bot64
F
Fpn
**
*
WT NT
WT LPS
cREL-/- NT
cREL-/- LPS

Figure S3. **Related to Figure 4**

(A) Mo-MACs of indicated genotypes were treated with LPS (100 ng/mL). 24 hr. later, RNA was extracted and the expression (normalized to *18S*) of *Fpn* measured by qRT-PCR. **(B and C)** Mo-MACs of indicated genotypes were treated with LPS (100 ng/mL). 24 hr. later, RNA was extracted and the expression (normalized to *18S*) of *Fpn* (**B**) and *Ho1* (**C**) measured by qRT-PCR. **(D)** Mo-MACs from WT mice were treated with LPS (1 µg/mL) with or without cycloheximide (10 µg/ml, added one hour before LPS). 8 hr. later, RNA was extracted and the expression (normalized to *Hprt*) of *Fpn* measured by qRT-PCR. **(E)** WT mice were treated (i.p) with LPS (single dose, 100 µg/mouse) with or without Bot64. Bot64 treatment (60 mg/Kg/day, three doses) was started 24 hr. before LPS treatment. 24 hr. after the final Bot64 treatment, mice were euthanized and lungs harvested. Shown is the expression (normalized to *Hprt*) of *Fpn* measured by qRT-PCR. **(F)** Mo-MACs from WT or cREL-knockout mice were treated with LPS (1µg/mL). 16 hr. later, RNA was extracted and the expression of *Fpn* (normalized to *Hprt*) measured by qRT-PCR.

qRT-PCR: data representative of $\geq$3 independent experiments and graphs show single experiment with n$\geq$2 per group. Results expressed as mean ± SEM. $P \leq 0.05$ (*), $P \leq 0.01$ (**), $P \leq 0.001$ (***), and $P \leq 0.0001$ (****).

Figure S4

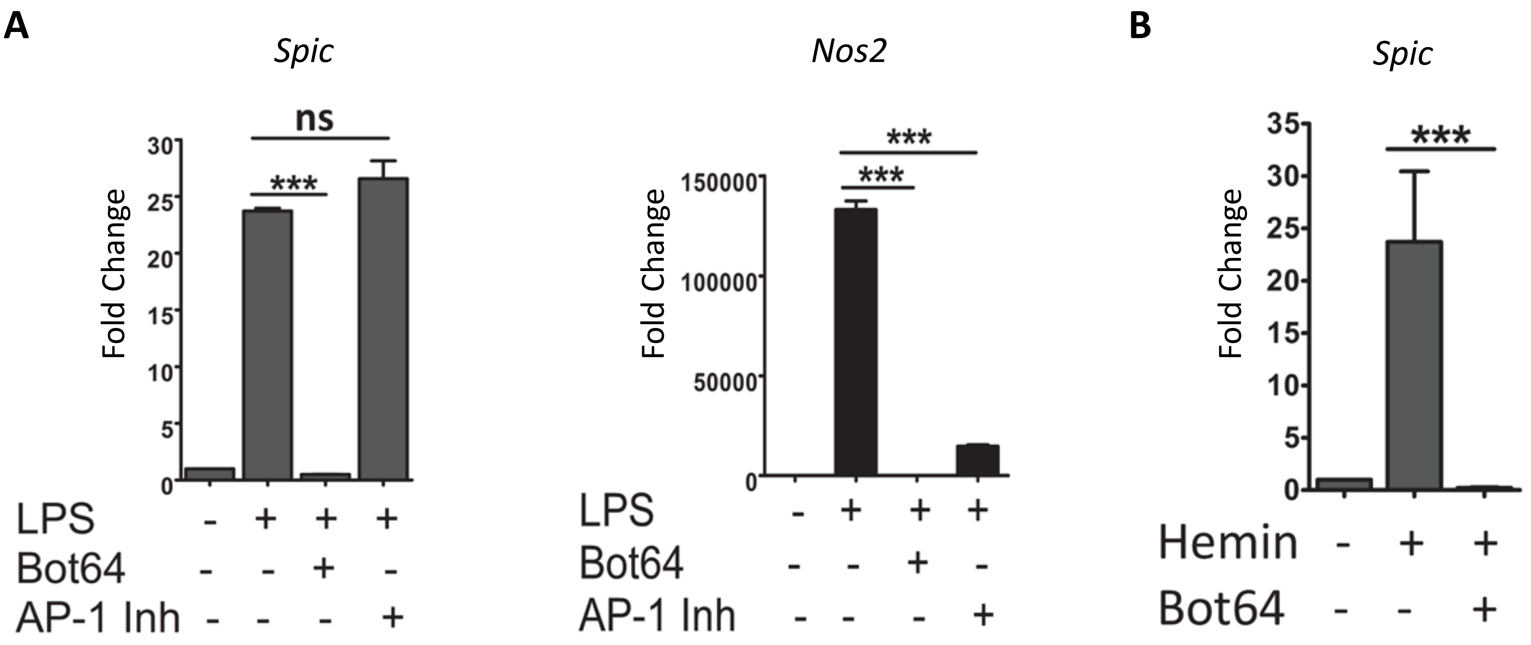
A
Spic
ns
***
Fold Change
30
25
20
15
10
5
0
LPS - + + +
Bot64 - - + -
AP-1 Inh - - - +
Nos2
***
***
Fold Change
150000
100000
50000
0
LPS - + + +
Bot64 - - + -
AP-1 Inh - - - +
B
Spic
***
Fold Change
35
30
25
20
15
10
5
0
Hemin - + +
Bot64 - - +

Figure S4. **Related to Figure 5**

(A) Mo-MACs were treated with LPS (1 µg/ml), IKK-2 inhibitor Bot64 (10 µM), or AP-1 inhibitor T5224 (10 µM). RNA was extracted 20 hr. later and the expression (relative to *Hprt*) of indicated genes (Y-axis, relative to no treatment group) measured by qRT-PCR. AP1 inhibitor blocked *Nos2* induction, but not *Spic*. **(B)** Mo-MACs from WT mice were treated with 80 µM hemin with or without IKK-2 inhibitor (Bot64, 10 µM). RNA was harvested 14 hr. later and the expression (normalized to *Hprt*) of *Spic* measured (Y-axis, relative to no treatment) by qRT-PCR. All qRT-PCR data shown is representative of ≥3 independent experiments. Plots show single experiment with n≥2 per group. To calculate the statistical significance for two individual groups, unpaired *t*-test were performed. To compare the mean of three or more groups, one-way ANOVA with Tukey's multiple comparison tests were used.

qRT-PCR: data representative of ≥3 independent experiments and graphs show single experiment with n≥2 per group. Results expressed as mean ± SEM. $P \leq 0.05$ (*), $P \leq 0.01$ (**), $P \leq 0.001$ (***), and $P \leq 0.0001$ (****).

Figure S5

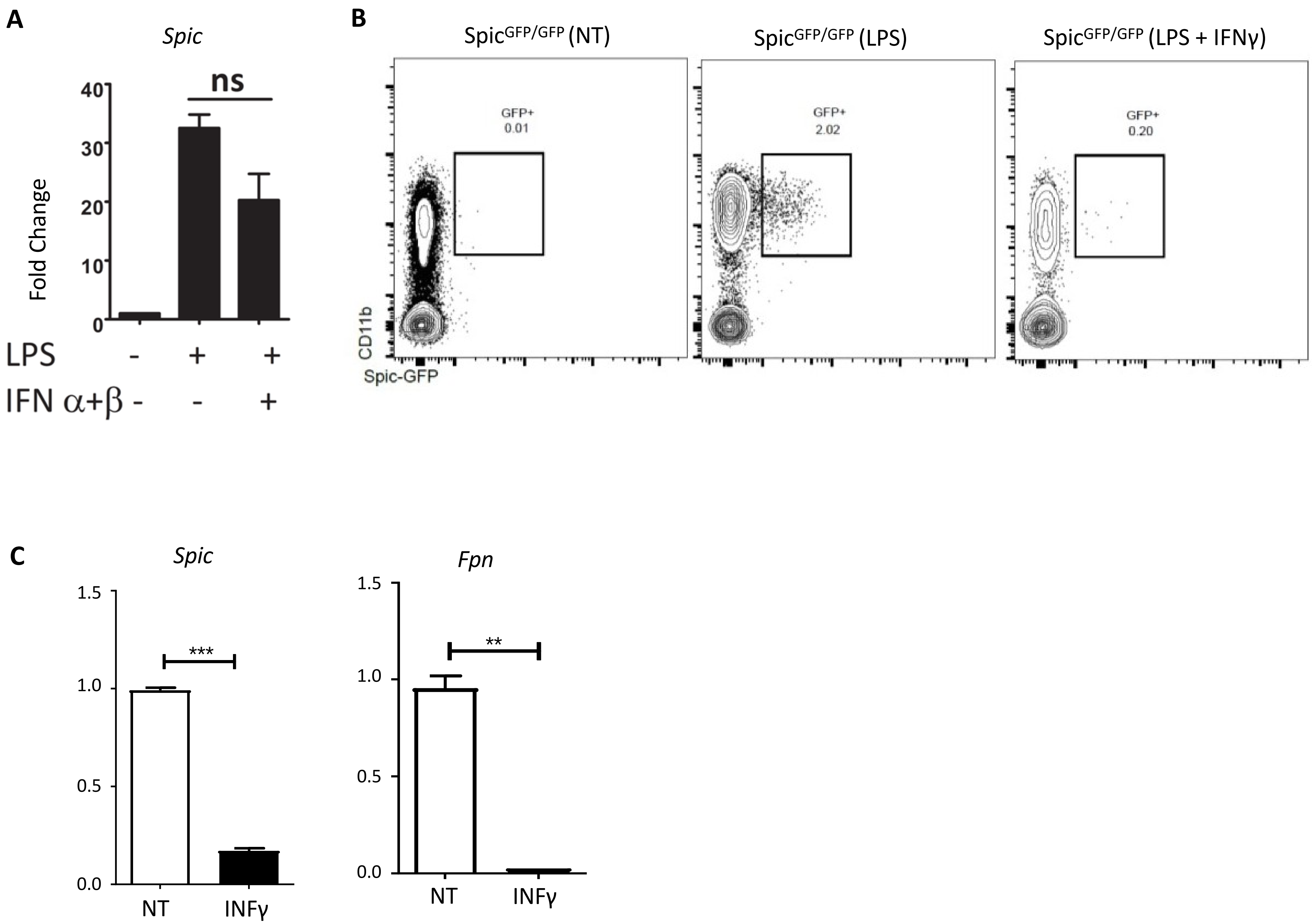
A
Spic
ns
Fold Change
40
30
20
10
0
LPS - + +
IFN α+β - - +
B
Spic^GFP/GFP (NT)
Spic^GFP/GFP (LPS)
Spic^GFP/GFP (LPS + IFNγ)
GFP+
0.01
GFP+
2.02
GFP+
0.20
CD11b
Spic-GFP
C
Spic
***
1.5
1.0
0.5
0.0
NT
INFγ
Fpn
**
1.5
1.0
0.5
0.0
NT
INFγ

Figure S5. Related to Figure 6

(A) BMDMs from WT mice were treated with LPS (1 μg/ml) with or without recombinant murine interferon-alpha (IFNα, 50 ng/ml) or interferon-beta (IFNβ, 50 ng/ml). RNA was extracted 18 hr. later and *Spic* expression (normalized to *Hprt*) were measured (Y-axis, fold change compared to no-treatment group) by qRT-PCR. (**B**) $Spic^{GFP/GFP}$ mice were treated with LPS (100μg/mouse) once with or without recombinant interferon-gamma (IFNγ, 20μg/mouse per injection for a total of five injections twice daily). Intraperitoneal IFNγ treatment was started a day before LPS injection. Peripheral blood was taken about 24 hr of LPS treatment. Shown are the flow cytometry plots with indicated markers on cells pre-gated for CD45+Ly6G- singlets. Numbers represent percentage of cells within indicated gate. Data represents 2 or more experiments with 3 or more mice per group. **(C)** BMDMs were treated with LPS (1μg/mL) with or without IFNγ, RNA extracted 20 hr. later, and the expression (normalized to *Hprt*) of *Spic* and *Fpn* measured (Y-axis) by qRT-PCR.

FCS: numbers represent percentage of cells within indicated gate. B represents 2 experiments with ≥3 mice per group. **qRT-PCR**: data representative of ≥3 independent experiments and graphs show single experiment with n≥2 per group. Results expressed as mean ± SEM. $P \leq 0.05$ (*), $P \leq 0.01$ (**), $P \leq 0.001$ (***), and $P \leq 0.0001$ (****).

Figure S6

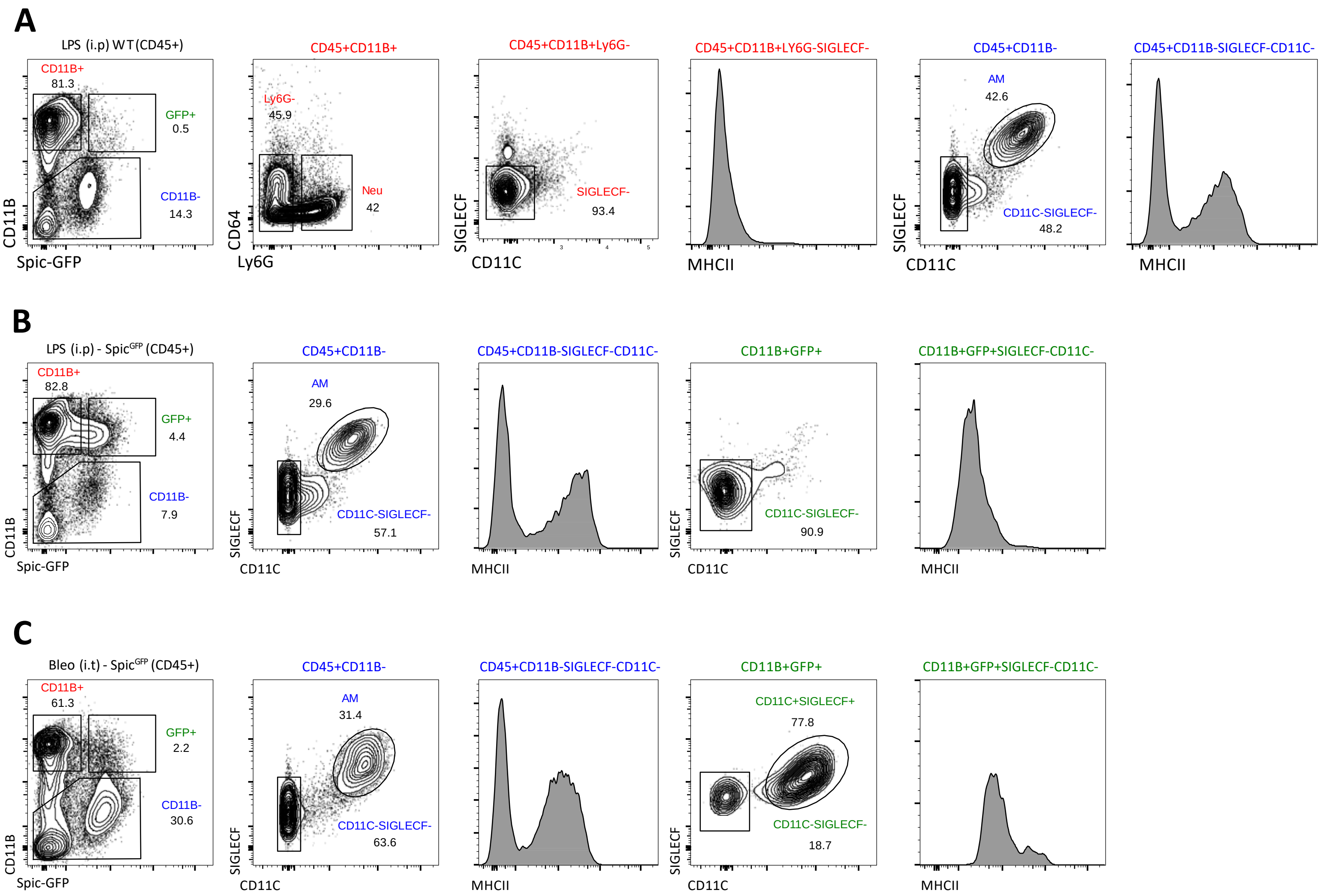
A
LPS (i.p) WT (CD45+)
CD11B+
81.3
GFP+
0.5
CD11B-
14.3
CD11B
Spic-GFP
CD45+CD11B+
Ly6G-
45.9
Neu
42
CD64
Ly6G
CD45+CD11B+Ly6G-
SIGLECF-
93.4
SIGLECF
CD11C
CD45+CD11B+LY6G-SIGLECF-
MHCII
CD45+CD11B-
AM
42.6
CD11C-SIGLECF-
48.2
SIGLECF
CD11C
CD45+CD11B-SIGLECF-CD11C-
MHCII
B
LPS (i.p) - Spic^{GFP} (CD45+)
CD11B+
82.8
GFP+
4.4
CD11B-
7.9
CD11B
Spic-GFP
CD45+CD11B-
AM
29.6
CD11C-SIGLECF-
57.1
SIGLECF
CD11C
CD45+CD11B-SIGLECF-CD11C-
MHCII
CD11B+GFP+
CD11C-SIGLECF-
90.9
SIGLECF
CD11C
CD11B+GFP+SIGLECF-CD11C-
MHCII
C
Bleo (i.t) - Spic^{GFP} (CD45+)
CD11B+
61.3
GFP+
2.2
CD11B-
30.6
CD11B
Spic-GFP
CD45+CD11B-
AM
31.4
CD11C-SIGLECF-
63.6
SIGLECF
CD11C
CD45+CD11B-SIGLECF-CD11C-
MHCII
CD11B+GFP+
CD11C+SIGLECF+
77.8
CD11C-SIGLECF-
18.7
SIGLECF
CD11C
CD11B+GFP+SIGLECF-CD11C-
MHCII

Figure S6. **Related to Figure 7 and 2**

(A) WT mice were treated (i.p) with LPS (150 µg/mouse), euthanized 48 hr. later, and their lungs analyzed by FCS using indicated markers. Headers denote pre-gating. The initial gates (first panel) are color-coded. Alveolar macrophages (AM, fifth panel) and the two subsets (MHC-II+ and MHC II-) of interstitial macrophages (IM, fifth and sixth panel) are clearly distinguishable. **(B)** *Spic*$^{GFP/GFP}$ reporter mice were treated (i.p) with LPS (150 µg/mouse) and euthanized 48 hr. later. Shown are the FCS plots of lungs using the gating strategy described in (A). AMs (second panel) and the two subsets of IMs (second and third panel) are clearly distinguishable. SPIC (GFP+) expressing cells (fourth and fifth panel) resemble MHCII+ IMs (fourth and fifth panel). **(C)** *Spic*$^{GFP/GFP}$ reporter mice were treated with intra-tracheal (i.t) bleomycin (3 U/kg in 50µl sterile PBS) and euthanized four weeks later. Shown are the FCS plots using the gating strategy described in (A). AMs (second panel) and the two subsets of IMs (third panel) are clearly distinguishable. In contrast to LPS treatment, SPIC (GFP+) expressing cells (fourth and fifth panel) appear to contain both AMs and the two subsets of IMs.

FCS: numbers represent percentage of cells within indicated gate. Data representative of ≥ 3 experiments with ≥ 3 mice per group per experiment.

Figure S7

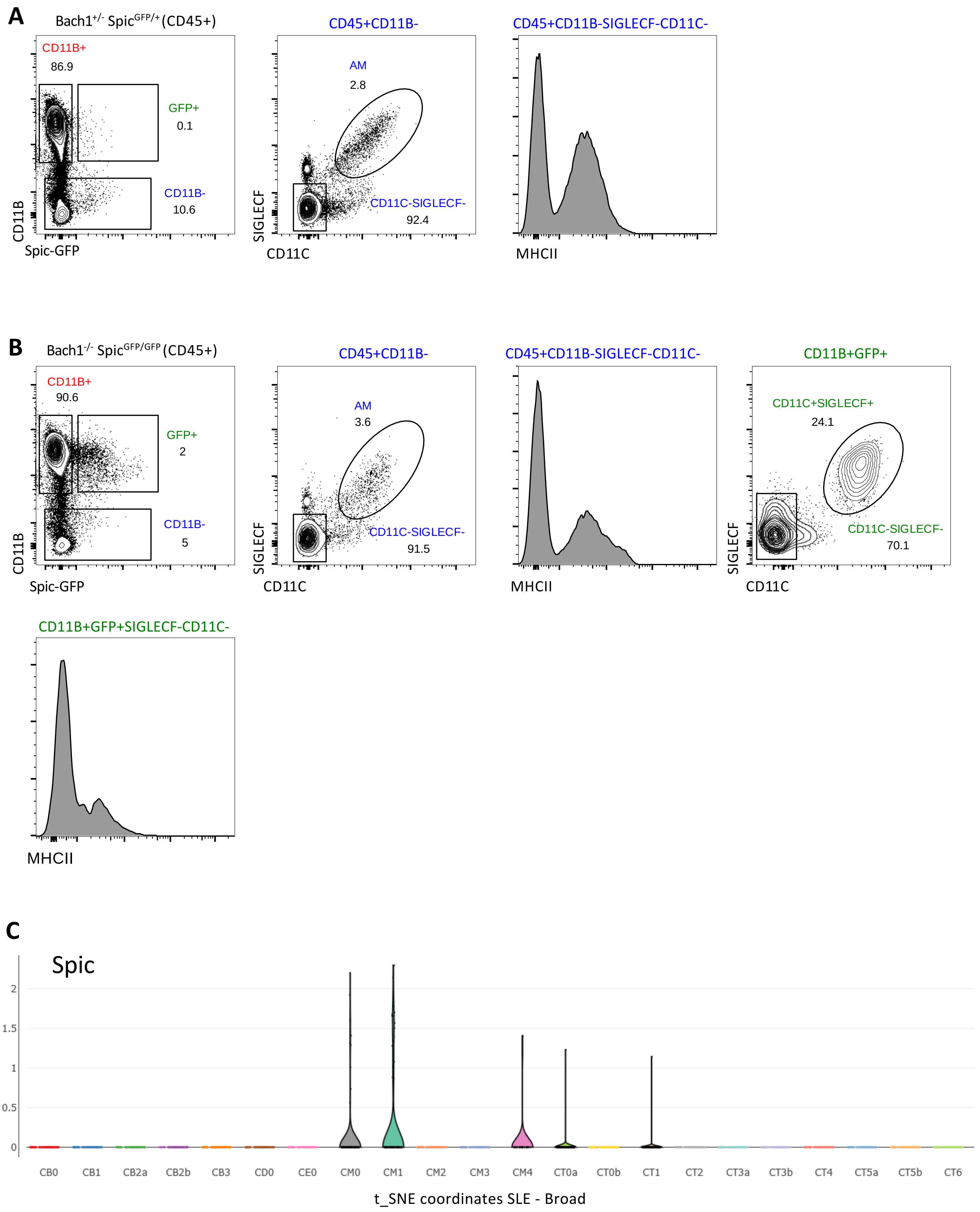
A
Bach1+/- SpicGFP/+ (CD45+)
CD11B+
86.9
GFP+
0.1
CD11B-
10.6
CD11B
Spic-GFP
CD45+CD11B-
AM
2.8
CD11C-SIGLECF-
92.4
SIGLECF
CD11C
CD45+CD11B-SIGLECF-CD11C-
MHCII
B
Bach1-/- SpicGFP/GFP (CD45+)
CD11B+
90.6
GFP+
2
CD11B-
5
CD11B
Spic-GFP
CD45+CD11B-
AM
3.6
CD11C-SIGLECF-
91.5
SIGLECF
CD11C
CD45+CD11B-SIGLECF-CD11C-
MHCII
CD11B+GFP+
CD11C+SIGLECF+
24.1
CD11C-SIGLECF-
70.1
SIGLECF
CD11C
CD11B+GFP+SIGLECF-CD11C-
MHCII
C
Spic
Expression
2
1.5
1
0.5
0
CB0
CB1
CB2a
CB2b
CB3
CD0
CE0
CM0
CM1
CM2
CM3
CM4
CT0a
CT0b
CT1
CT2
CT3a
CT3b
CT4
CT5a
CT5b
CT6
t_SNE coordinates SLE - Broad

Figure S7. Related to Figure 7 and 2

(A) Lungs from *Bach1*$^{+/-}$ *Spic*$^{GFP/+}$ mice were analyzed by FCS using the indicated markers. The gates are color-coded and very few GFP + *Spic*-expressing cells are seen (first panel). AMs (middle panel) and the two subsets of IMs (last panel) are identified.

(B) Lungs from *Bach1*$^{-/-}$ *Spic*$^{GFP/GFP}$ mice were analyzed by FCS using the indicated markers. The gates are color-coded and highlights the presence of GFP+ *Spic*-expressing cells in the absence of *Bach1* (first panel). AMs (second panel) and the two subsets of IMs (third panel) are identified. SPIC (GFP+) expressing cells (fourth and fifth panel) appear to contain both AMs and the two subsets of IMs.

FCS: numbers represent percentage of cells within indicated gate. Data representative of ≥ 3 experiments with ≥ 3 mice per group per experiment

(C) The expression of Spic was examined in single cell RNA-sequencing dataset from Kidneys of normal humans and patients of lupus nephritis (Arazi et al., 2019). This data was generated using the freely available single cell portal from the broad institute (https://singlecell.broadinstitute.org/single_cell/study/SCP279/amp-phase-1) using their lupus nephritis dataset. Shown is the expression of Spic (Y-axis) in various kidney-infiltrating immune cells (X-axis).

As described in detail by Arazi and colleagues, cluster CM0 to CM4 were identified as myeloid cells. CM0, CM1, and CM4 were identified as kidney-infiltrating monocytes and macrophages as these cells were enriched in nephritic kidneys compared to normal kidneys. Of these, CM1 and CM4 were identified as phagocytic macrophages. CM4 was further characterized as alternatively activated macrophages with high expression of ferroportin. Further analyses by Arazi and colleagues uncovered a differentiation trajectory where blood monocytes (CM0) differentiate into phagocytic monocyte/macrophage (CM1), which further differentiates into an alternatively activated macrophage (CM4) in nephritic kidneys (Arazi et al., 2019). In contrast, CM2 was the dominant myeloid cells in normal kidneys and characterized as tissue-resident macrophages (Arazi et al., 2019). The relatively specific expression of Spic within the inflammation-associated monocyte-derived lineage (CM0, CM1, and CM4), but not in dendritic cells (CM3) or tissue-resident macrophages (CM2), is consistent with our observations in murine inflammation models.

CM0: Patrolling CD16+ macrophages, CM1: Phagocytic CD16+ macrophages, CM2: Tissue-resident macrophages, CM3: cDCs, CM4: M2-like CD16+ macrophages, CT0a: Effector memory CD4+ T cells, CT0b: Central memory CD4+ T cells, CT1: CD56dimCD16+ NK cells, CT2: CTLs, CT3a: Treg cells, CT3b: TFH-like cells, CT4: GZMK+ CD8+ T cells, CT5a: Resident memory CD8+ T cells, CT5b: CD56brightCD16– NK cells, CT6: ISG-high CD4+ T cells, CB0: Activated B cells, CB1: Plasma, cells and plasmablasts, CB2a: Naive B cells, CB2b: pDCs, CB3: ISG-high B cells, CD0: Dividing cells, CE0: Epithelial cells.
